# Distinct Valence States of the [4Fe4S] Cluster Revealed in the Hydrogenase *Cr*HydA1

**DOI:** 10.1002/anie.202424167

**Published:** 2025-02-05

**Authors:** Melanie Heghmanns, Shalini Yadav, Sergius Boschmann, Victor R. Selve, Astrit Veliju, Claudia Brocks, Thomas Happe, Dimitrios A. Pantazis, Müge Kasanmascheff

**Affiliations:** ^1^ Department of Chemistry and Chemical Biology TU Dortmund University Otto-Hahn-Strasse 4a 44227 Dortmund Germany; ^2^ Max-Planck-Institut für Kohlenforschung Kaiser-Wilhelm-Platz 1 45470 Mülheim an der Ruhr Germany; ^3^ Faculty of Biology and Biotechnology Photobiotechnology Ruhr-University Bochum Universitätsstrasse 150 44801 Bochum Germany

**Keywords:** Metalloproteins, [FeFe]-hydrogenase, Iron-sulfur cluster, Valence isomerism, EPR spectroscopy

## Abstract

Iron‐sulfur clusters play a crucial role in electron transfer for many essential enzymes, including [FeFe]‐hydrogenases. This study focuses on the [4Fe4S] cluster ([4Fe]_H_) of the minimal [FeFe]‐hydrogenase from *Chlamydomonas reinhardtii* (*Cr*HydA1) and employs advanced spectroscopy, site‐directed mutagenesis, molecular dynamics simulations, and QM/MM calculations. We provide insights into the complex electronic structure of [4Fe]_H_ and its role in the catalytic reaction of *Cr*HydA1, serving as paradigm for understanding [FeFe]‐hydrogenases. We identified at least two distinct species within the apo‐form of *Cr*HydA1, designated 4Fe−R and 4Fe−A, with unique redox potentials and pH sensitivities. Our findings revealed that these species arise from a complex interplay of structural heterogeneity and valence isomer rearrangements, influenced by second‐sphere residues. We propose that the interconversion between 4Fe−R and 4Fe−A could provide control over electron transfer in the absence of accessory FeS clusters typically found in other [FeFe]‐hydrogenases. The insights gained from this study not only enhance our understanding of [FeFe]‐hydrogenases but also provide a crucial foundation for future investigations into analysis of other FeS clusters across diverse biological systems.

## Graphical abstract

In the apo‐form of FeFe‐hydrogenase from *Chlamydomonas reinhardtii* two different paramagnetic states arise from a single iron‐sulfur cluster. EPR experiments found them to interconvert based on redox‐potentials and pH‐levels. Computational methods attribute this to secondary‐sphere residues adopting distinct conformations around the cluster.

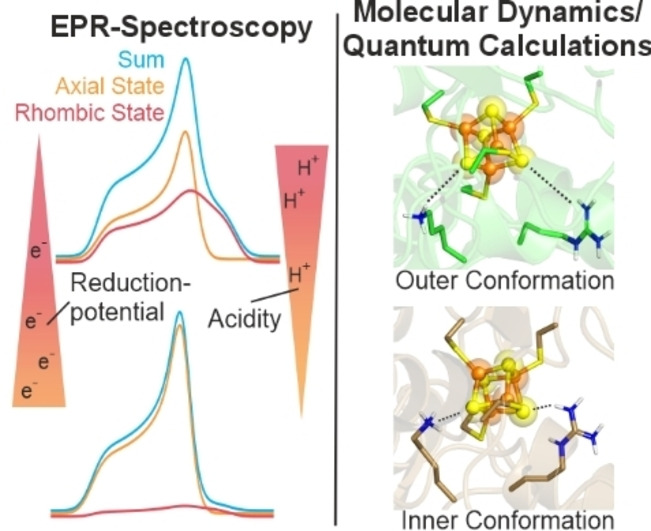

## Introduction

**Figure 1:**
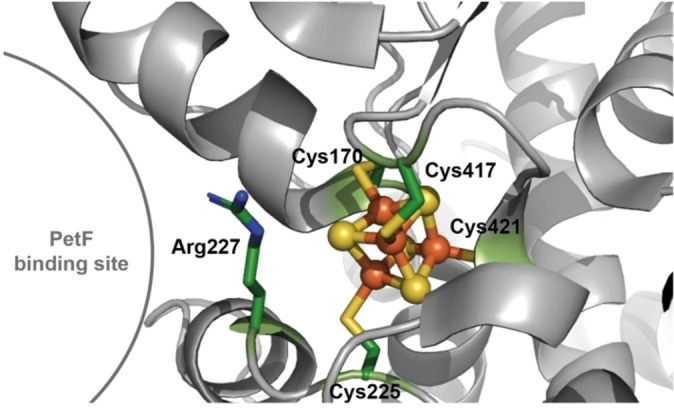
(**A**) Crystal structure (grey) of apo‐*Cr*HydA1 (PDB ID: 3LX4) displaying the [4Fe4S] cluster ([4Fe]_H_) as sticks and spheres. The coordinating cysteine ligands (Cys170, Cys225, Cys417, and Cys421) and residue Arg227 are shown as sticks. Color coding: Fe: orange, S of [4Fe]_H_ and the ligating cysteines: yellow, C: green, N: blue).

[FeFe]‐hydrogenases perform a seemingly simple yet crucial task: the reversible reduction of protons to molecular hydrogen.^[1]^ These enzymes, found across diverse organisms, display a high modular diversity including varying domains and accessory iron‐sulfur (FeS) clusters.^[2]^ Among these molecular machines, HydA1 from the green alga *Chlamydomonas reinhardtii* (*Cr*HydA1) stands out for its simplicity and is one of the best characterized [FeFe]‐hydrogenases.[^3^, ^4^, ^5^, ^6^] Unlike its bacterial counterparts, *Cr*HydA1 consists solely of the H‐domain only,[^3^, ^7^, ^8^] housing the active site known as the H‐cluster. This cluster, the engine of hydrogen production, features a cubane [4Fe4S] cluster, [4Fe]_H_, connected to an electronically coupled dinuclear cluster, [2Fe]_H_.[^9^, ^10^] The dinuclear cluster, coordinated by inorganic ligands and bridged by an azadithiolate (adt) ligand, has an open coordination site where catalysis occurs.^[6]^ The protons are transferred via a specific amino acid‐based pathway from the enzyme's surface to the amine group of the adt ligand.[^11^, ^12^, ^13^] The [4Fe]_H_ subsite serves as the electron gateway and its redox potential is suggested to influence the catalytic bias.[^14^, ^15^] Under physiological conditions, *Cr*HydA1 receives electrons from PetF, a [2Fe2S] cluster‐containing ferredoxin.^[16]^ A second sphere residue, Arg227 in *Cr*HydA1, has been reported to be crucial for the efficient electron transfer from PetF to *Cr*HydA1 (Figure 1).[^17^, ^52^]

While the structure of *Cr*HydA1 with its intact H‐cluster remains elusive, significant insights have been gained from studying its apo‐form. The crystal structure of apo‐*Cr*HydA1 (Figure 1) (PDB code: 3LX4^[8]^) revealed an empty cavity for the usual location of [2Fe]_H_. Residual density at this empty site was modeled with an acetate molecule and chloride ion.^[8]^ Spectroscopic evidence from electron paramagnetic resonance (EPR) and Mössbauer studies verified [4Fe]_H_ being the only FeS cluster present.[^8^, ^18^] The *g*‐values observed for the reduced [4Fe]_H_ in EPR spectra of apo‐*Cr*HydA1 align with the typical range for reduced [4Fe4S]^1+^ clusters with a spin state of *S*=1/2.[^19^, ^20^, ^21^, ^22^, ^23^] Intriguingly, there exists a notable discrepancy in the reported *g*‐values for [4Fe]_H_ across different research groups (Table 1). The observed variations in *g*‐values, which are the most characteristic parameters – often considered the ‘fingerprint’ of a spin system –, are unlikely to stem from different measurement techniques. This raises important questions about the factors influencing these unexpected differences and their molecular origin.

**Table 1:** List of *g*‐values reported in the literature for the EPR spectrum of the [4Fe4S]^1+^ cluster of apo‐*Cr*HydA1 obtained with distinct methods.

*g*‐values			Method	Reference
2.040	1.910	1.910	X‐band cw EPR	[18]
2.045	1.926	1.896	Q‐band FID‐detected EPR	[29]
2.050	1.915	1.852	Q‐band ESE‐detected EPR	[33]
2.054	1.921	1.848	Q‐band ESE‐detected EPR	[34]

The electronic structure of a reduced [4Fe4S]^1+^ cluster with a *S* = 1/2 ground state can be pictured as two Fe^2+^ ions, which are antiferromagnetically coupled to a delocalized mixed‐valence Fe^2.5+^ pair.[^21^, ^24^, ^25^] In turn, six energetically inequivalent electronic states are formed by different arrangements of the four Fe ions, known as valence isomers.[^26^, ^27^] These valence isomers each experience a slightly distinct, asymmetric protein environment and exhibit each their own EPR spectrum having slightly distinct *g*‐values.^[27]^ Such observations were made for [4Fe4S]^3+^ clusters and synthetic [4Fe4S]^1+^ clusters.[^26^, ^27^, ^28^] Changes in the first coordination sphere of [4Fe4S] clusters were shown to impact the distribution of valence isomers.^[27]^

[4Fe]_H_ is ligated by four cysteine residues (C170, C225, C417, and C421; Figure 1), whereby the first is suggested to be essential for stable cluster integration and the latter for [2Fe]_H_‐anchoring.^[29]^ Modification of these cysteine residues revealed changes in electronic and catalytic properties.[^15^, ^29^] This underlines the strong synergy between the electronic structure of [4Fe]_H_ and the protein scaffold. Since [4Fe]_H_ is redox‐active^[10]^ and required for a successful [2Fe]_H_‐integration,^[18]^ a detailed investigation and understanding of the [4Fe]_H_‐site is particularly relevant for future research on minimal hydrogenases.[^30^, ^31^, ^32^] Yet, the electronic coupling and overlapping signatures of the [4Fe]_H_ and [2Fe]_H_ clusters pose challenges for their detailed study. Therefore, studying apo‐*Cr*HydA1 offers unique opportunities to obtain highly specific insights on [4Fe]_H_.

Here, we investigated apo‐*Cr*HydA1 under various conditions with spectroscopic and computational techniques. We detected the presence of more than one paramagnetic species arising from [4Fe]_H_ using multi‐frequency, variable‐temperature, and potential‐dependent EPR spectroscopy. Molecular dynamics (MD) simulations and quantum mechanics/molecular mechanics (QM/MM) calculations showed valence isomer rearrangements of [4Fe]_H_ upon flexibility of the second‐sphere residues. We propose that the detected species dynamically control electron entry to the H‐cluster. Our findings reconcile previously reported variabilities in *g*‐values for [4Fe]_H_ and provide novel insights into the electronic structure of this FeS cluster and its interplay with the protein scaffold. These results significantly contribute to our understanding of minimal hydrogenases and potentially inform future bioengineering efforts aimed at optimizing hydrogen production.

## Results and Discussion

### Observation of Two Distinct Species for the [4Fe4S]^+^ Cluster of apo‐CrHydA1

**Figure 2:**
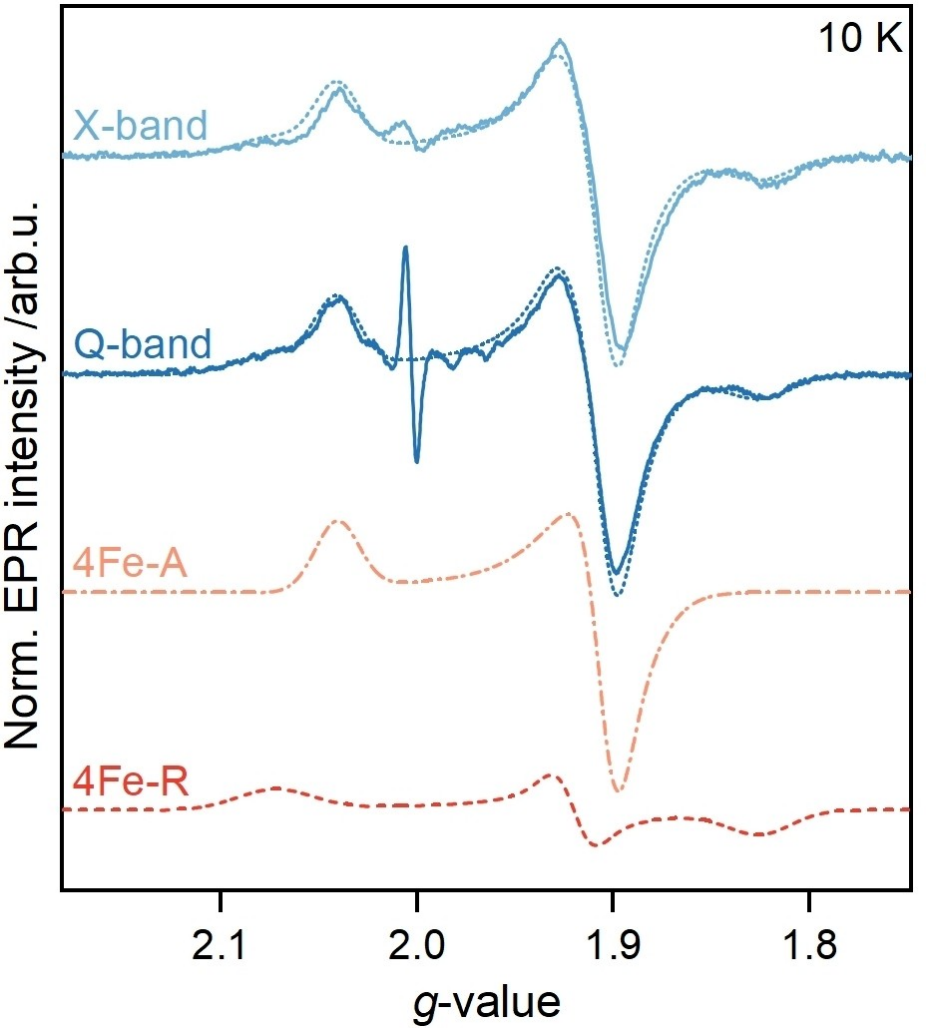
Comparison of ESE‐detected EPR spectra (9.7 and 34 GHz, *T* = 10 K, solid traces) of apo‐*Cr*HydA1 reduced with 10 mM NaDT in TIP buffer shown as pseudo‐modulations (1.4 and 4 mT modulation amplitude). The full spectral simulations (dotted traces) comprise the axial species 4Fe‐A (dashed orange trace) and the rhombic species 4Fe‐R (red dashed traces).

To reassess [4Fe]_H_ in apo‐*Cr*HydA1, we first recorded temperature‐dependent EPR spectra of the dithionite‐reduced enzyme (Figure 2 and S2). At temperatures above 20 K the EPR signal was undetectable due to spectral line broadening. This temperature‐dependent behavior demonstrates the presence of a fast‐relaxing [4Fe4S]^+^ cluster and the absence of slower‐relaxing species, such as [2Fe2S] clusters, aligning well with the literature.^[18]^

The EPR spectrum of apo‐*Cr*HydA1 reveals a rhombic line shape similar to previously observed spectra.[^18^, ^29^, ^33^, ^34^] Spectral simulations with a single species could not reproduce the experimental data. An excellent simulation, however, is achieved with two distinct *S* = 1/2 species: an axial and a rhombic species, termed 4Fe−A and 4Fe−R, respectively (see Table S1 for *g*‐values). Both species are present in nearly equal amounts, ruling out a minor species arising from contamination (Figure 2). The *g*‐values agree well with [4Fe4S]^1+^ clusters.[^19^, ^20^, ^21^, ^22^] Notably, the results were reproducible across different batches of sample preparations (Figure S2 and Table S1). Interestingly, the *g*_2_≈1.90 value detected for both species deviates from the usual observed *g*_2_≈1.94 in all‐cysteine ligated clusters.[^35^, ^36^] Although *g*_2_≈1.90 is attributed to [4Fe4S]^1+^ clusters with non‐cysteine ligands, this is not supported by the crystal structure of apo‐*Cr*HydA1.^[8]^

The crystal structure shows that the only metallocluster in apo‐*Cr*HydA1 is [4Fe]_H_. However, the absence of the neighboring [2Fe]_H_ leaves a water‐filled cavity of 10 Å diameter,[^8^, ^37^] potentially hosting additional interacting species. Magnetic interactions between them may result in exchange coupling, manifesting as a frequency‐dependent line shape, broadened features, or anomalous *g*‐values.[^38^, ^39^, ^40^] Therefore, we used multi‐frequency EPR to probe for magnetic exchange interactions between paramagnetic centers within 15 Å of each other.^[41]^ The comparison of apo‐*Cr*HydA1 spectra recorded at X‐ and Q‐band frequencies showed no significant change in *g*‐values and the line shape (Figure 2). This finding excludes spin‐spin interactions as the source of the rhombic features, here assigned to species 4Fe‐R. Furthermore, we did not detect any features in the low‐field region of the X‐ and Q‐band EPR data, excluding the presence of *S*> 1/2 species observed for some [4Fe4S]^1+^ clusters,[^21^, ^42^] or half‐field transitions arising from exchange interactions.[^43^, ^44^]

Despite reports that apo‐*Cr*HydA1 primarily exists as monomers,^[45]^ we investigated whether the broad features associated with 4Fe−R are derived from two interacting [4Fe]_H_ clusters located within a multimer of apo‐*Cr*HydA1 by conducting distance measurements using double electron‐electron resonance (DEER) spectroscopy. The resulting DEER trace revealed no dipolar modulations (Figure S3), strongly indicating the absence of two interacting [4Fe]_H_ clusters within a distance of 1.5 nm up to 8 nm in separate monomers.[^46^, ^47^]

We next investigated the effect of distinct sample preparations and measurement conditions on 4Fe‐A and 4Fe‐R (Figure S4). With varying buffer compositions and dithionite concentrations the rightmost feature, associated with 4Fe‐R, was slightly altered, highlighting the rhombic species’ sensitivity to variations in sample preparations. But overall, no significant changes in the EPR line shape were observed.

### Potentiometric Titration and pH‐dependent Measurements Enable the Separation of Two Species

**Figure 3:**
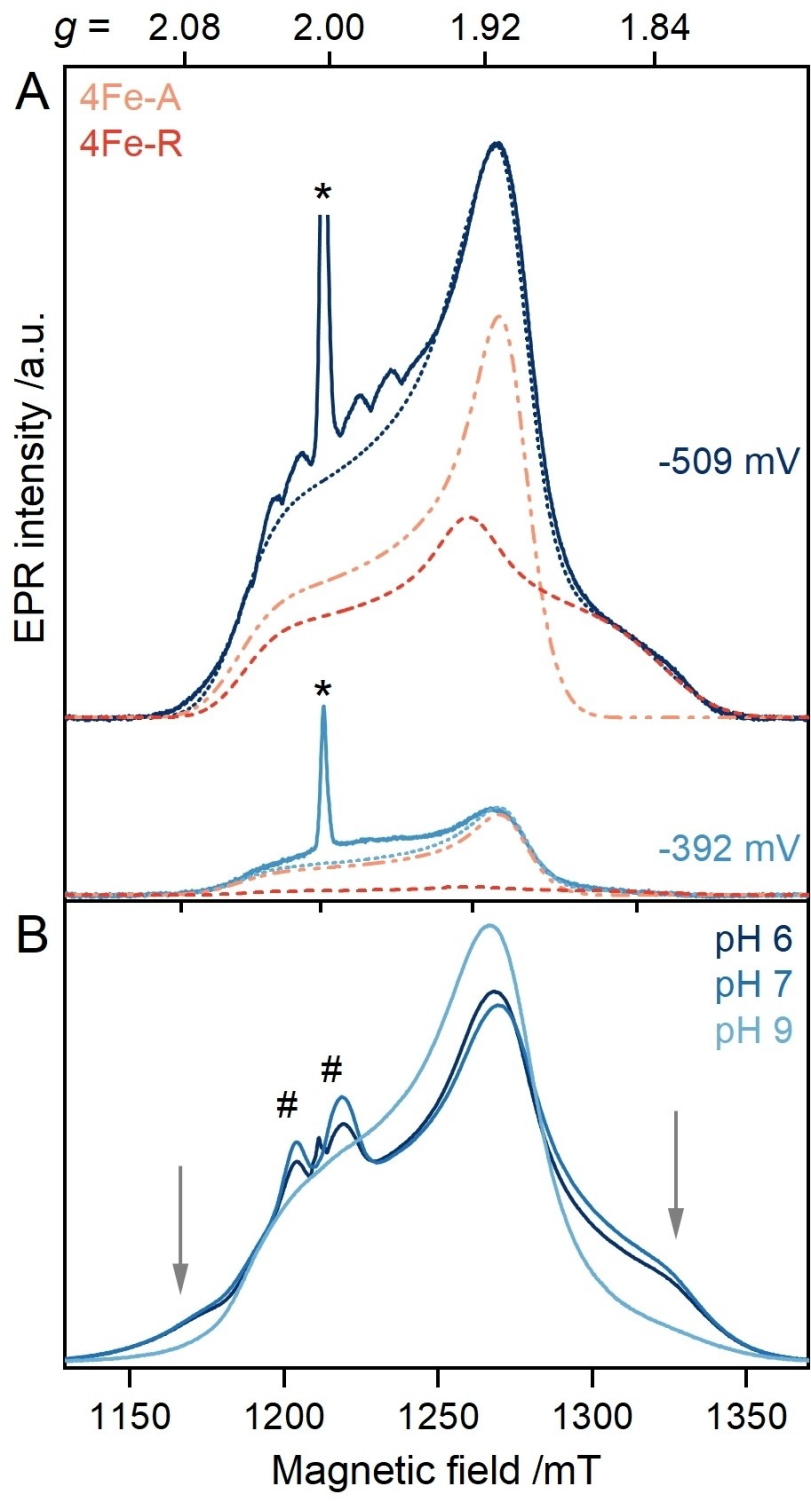
Comparison of ESE‐detected EPR spectra (34 GHz, *T* = 8/10 K, solid traces) of apo‐*Cr*HydA1 (**A**) at indicated potentials or (**B**) in buffers with distinct pH values and 5 mM NaDT. The spectral simulations of 4Fe−A (dotted orange trace) and 4Fe−R (dashed red trace) yield the full spectral simulation (dotted blue trace). The respective weights used for the total simulations are reported in Table S2. An organic radical marked with * is cut off for better visualization. Unusual features, attributed to an exchange coupled organic radical, are marked by # (see Figure S6). The grey arrows mark 4Fe−R present in the pH 6/7 samples of apo‐*Cr*HydA1.

To deconvolute the two species, EPR spectra of apo‐*Cr*HydA1 were recorded as a function of varying potentials (Figure 3 and S5). A sample recorded at *E*=−509 mV (*vs*. SHE) revealed a mixture of 4Fe−A and 4Fe−R with an almost equal amount (Figure 3 and Table S2). Upon increasing potentials, the intensity of 4Fe−R decreases and at *E*=−392 mV (*vs*. SHE) mainly 4Fe−A is detectable (Figure 3). These results confirm the presence of two discernible species with distinct redox potentials, which allow for a potential‐dependent separation. We refrain from determining the midpoint potentials at this point due to the complex temperature dependence and relaxation behavior of both species (see below).

Next, we investigated the possibility of pH‐dependent formation of these two distinct species. Indeed, the EPR spectra of reduced apo‐*Cr*HydA1 at pH 6–7 show a 1 : 2 mixture of 4Fe−A and 4Fe−R, whereas at pH 9 mainly 4Fe−A is detectable (Figure 3). This observation suggests a pH‐dependent formation of 4Fe−R. Additional features at *g*≈2 observable at low pH are attributed to an exchange coupled organic radical (see Figure S6 and accompanying discussion). Despite differing pH, the EPR signal intensities remained similar. This indicates (i) no degradation, cluster loss, or formation of a diamagnetic species and (ii) a direct conversion between species. Moreover, the pH‐dependent changes indicate protonation differences in the vicinity of [4Fe]_H_ associated with 4Fe−R.

Overall, the potential‐ and pH‐dependent spectra verify the presence of two [4Fe]_H_ species having distinct electronic structures and slightly changed proton environments in apo‐*Cr*HydA1.

### Variable‐temperature EPR Spectra Unveil Various Overlapping Species

**Figure 4:**
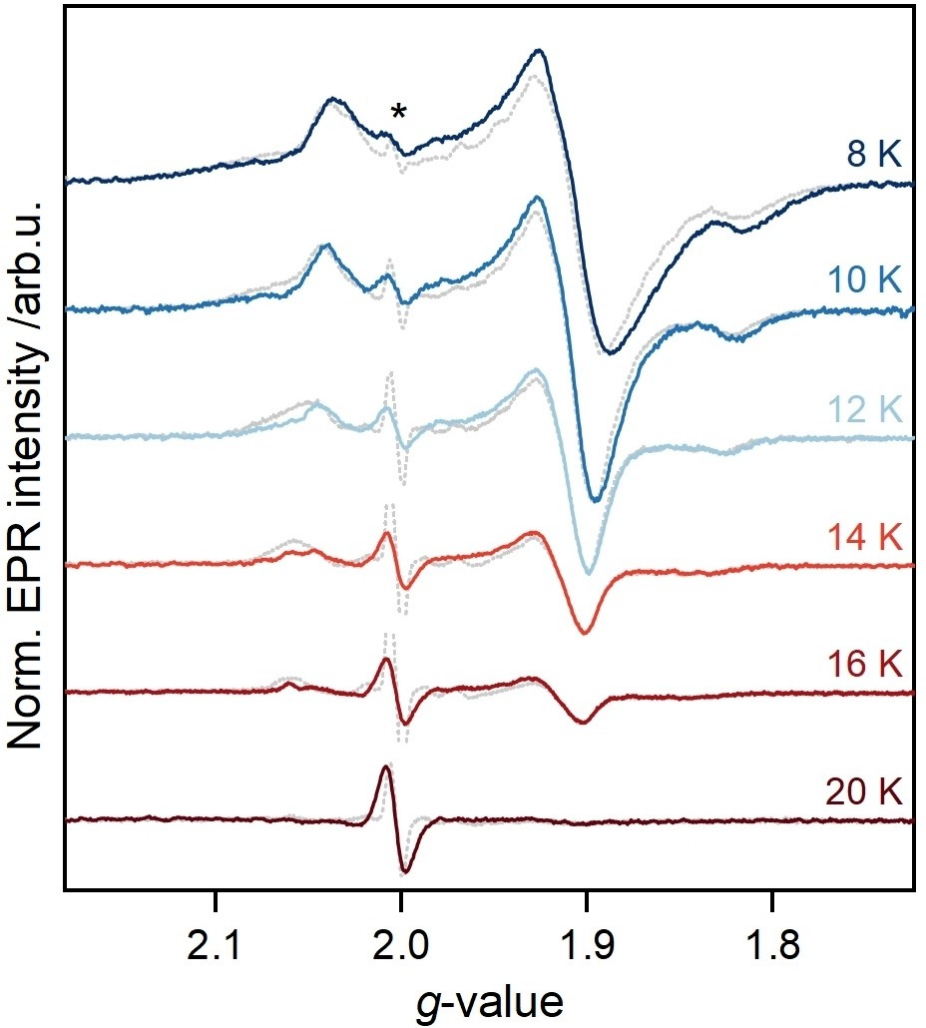
Comparison of temperature‐dependent, ESE‐detected EPR spectra of apo‐*Cr*HydA1 in TIP buffer at pH 7.5 reduced with 10 mM NaDT recorded and pseudo‐modulated at 9.7 GHz (colored traces, normalized for temperature) and 34 GHz (grey dotted traces). The Q‐band spectra were normalized to the X‐band spectra at the respective temperature. An organic radical signal is marked with * and was cut off, if necessary, for better visibility.

Our variable‐temperature pulsed EPR experiments at different frequencies are depicted in Figure 4 and S7. After careful comparison, we observed a slight change in the EPR line shape with increasing temperature. To investigate whether this phenomenon is exclusive to apo‐*Cr*HydA1, we recorded variable‐temperature EPR spectra of a plant‐type ferredoxin carrying one [2Fe2S] cluster with defined *g*‐values (Figure S8). This protein represents an effective model to assess FeS cluster properties as it is small (ca. 10 kDa) and soluble.^[50]^ Interestingly, we observed a similar temperature‐dependent behavior of the [2Fe2S] cluster, suggesting that this observation may not only be restricted to [4Fe]_H_ but is generally detectable for at least some types of FeS clusters.

Next, we investigated whether the observed effect is due to the nature of the pulsed EPR method. To this end, we recorded variable‐temperature continuous wave (cw) EPR spectra with apo‐*Cr*HydA1 (Figure S9). The changes in line shape upon temperature are almost undetectable in these spectra compared to pulsed experiments. One possible explanation for this discrepancy between cw and pulsed EPR spectra is the relative insensitivity of cw EPR to relaxation times, which are strongly dependent on the electronic structure of the paramagnetic species (see Figure S10 and associated discussion).^[51]^ Based on these results, we concluded that the observed effect is not a method and/or sample‐dependent artifact.

Building on previous work that demonstrated the existence of multiple [4Fe4S]^3+^ forms, attributed to valence isomers of a single cluster,^[28]^ we adopted a similar strategy in our analysis. We incorporated up to eight species with *g*‐values differing subtly from those of 4Fe−A and 4Fe−R into our simulations. This method yielded an exceptionally close match to our multi‐frequency EPR data (Figure S11–12 and Table S3). Notably, we observed that the number of discernible species increased as the temperature decreased. This temperature‐dependent behavior strongly supports the presence of multiple species, each with distinct relaxation properties.

It is important to emphasize that the complexity of this system introduces numerous variables, making a definitive analysis challenging. Our simulations incorporating eight species serve primarily as an illustrative tool, demonstrating the potential for multiple species. While these simulations achieve a close fit with experimental data, they should not be interpreted as providing exact *g*‐values, species counts, or relative proportions. Rather, they offer a plausible model that aligns with our observations and supports the concept of multiple, slightly distinct electronic states within [4Fe]_H_.

### Conformational Heterogeneity Revealed by Molecular Dynamics and Quantum Mechanics/Molecular Mechanics Calculations

To obtain insight into how the protein environment and its conformational flexibility might influence the electronic structure of [4Fe]_H_, we constructed all‐atom computational models of a protein monomer, conducted MD, as well as QM/MM optimizations of the [4Fe]_H_ and its immediate environment using selected MD snapshots.

**Figure 5:**
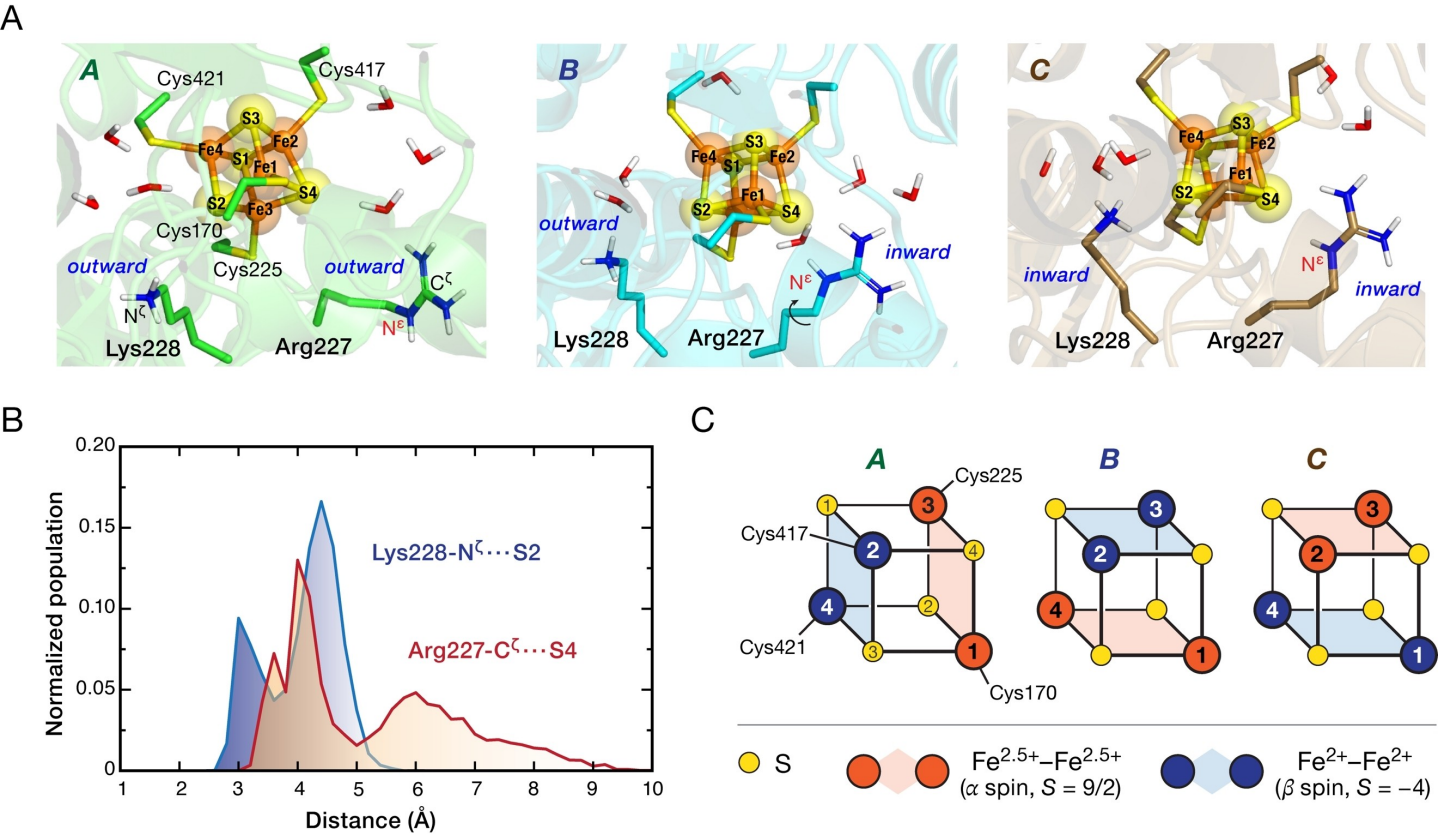
(**A**) Visual depiction of three major conformations observed during MD simulations, defined by the “inward” or “outward” orientation of the Arg227 and Lys228 side chains with respect to the [4Fe]_H_ cluster. These reorientations are accompanied by longer‐scale rearrangements of the second coordination sphere of the cluster. (**B**) Distribution of distances between the Arg227 side chain C^ζ^ atom and the S4 bridge, as well as between the Lys228 side chain N^ζ^ atom and the S2 bridge, showing the distinct distribution profiles for the major “inward” and “outward” configurations of the two residues. (**C**) Schematic depiction of the valence isomers obtained by QM/MM calculations on the three types of configurations.

Analysis of the MD trajectory reveals a highly dynamic second coordination sphere affecting the local environment of [4Fe]_H_. Figure 5 highlights the two closest second‐sphere residues, Arg227 and Lys228, which can adopt conformations facing either ‘inward’ toward the cluster or ‘outward’, away from the cluster. We note in passing that the acetate and chloride ions depart from the cavity very early in the MD run, suggesting adventitious binding in the crystal. Among the many conformations that are sampled by MD, three distinct and most populated combinations during the MD have been singled out to further explore the influence of the second coordination sphere flexibility on the valence states of [4Fe]_H_. These are shown in Figure 5 as conformations *A*, *B*, and *C*.

In *A*, the side chains of both residues (Arg227 and Lys228) face outward from the cluster while in *C* both face inward. Distance plots and population analyses (Figure S13) show that both residues span a wide range of orientations as they are not locked in place by specific hydrogen bonding interactions. Both residues exhibit frequent fluctuations between inward and outward positions (Figure S13) with distinct broadness of their distance distributions (Figure 5B) yet with clear distinction between inward and outward facing regions. In the case of ‘inward’ orientations, the guanidium side chain of Arg227 is positioned at a hydrogen‐bonding distance to the S4 sulfur bridge and the Cys225 ligand of [4Fe]_H_ (in approximately a third of the MD trajectory), while Lys228 is at a hydrogen‐bonding distance from the S2 bridge. It is noted that the position of the N^ϵ^H (in red) in conformers *A* versus *B*/*C* (Figure 5) indicates that the two guanidinium conformations are switched by flipping –not shifting– of the Arg side chain. In conclusion, we identified two flexible second sphere residues, of which one (Arg227) is crucial for the catalytic activity of *Cr*HydA1,^[52]^ that interact with the active site [4Fe]_H_ via distinct conformational changes.

Nevertheless, we stress that the conformational profile of the [4Fe]_H_ environment is not uniquely defined by the orientation of these two residues. This is due to the fact that each intermediate conformation of the two residues and each mutual combination are accompanied by movements of water molecules and reorganization of the hydrogen‐bonding network in the vicinity of the cluster (Figure S14). This shows that multiple structural elements contribute to the fluxionality of this cluster.

Importantly, the arrangement of these two second‐sphere residues has a marked effect on the valence distribution of the cluster. It is known that the valence distribution within [4Fe4S]^1+^ clusters is influenced by the composition and alterations of their primary coordination sphere.[^27^, ^53^, ^54^, ^55^, ^56^, ^57^, ^58^, ^59^] To examine whether the observed flexibility of the *second* sphere residues identified above impact the electronic properties of [4Fe]_H_, we performed QM/MM calculations with ORCA,^[60]^ using the ZORA scalar relativistic Hamiltonian^[61]^ with the B3LYP functional[^62^, ^63^] and all‐electron ZORA‐recontracted TZVP basis sets[^64^, ^65^] for the QM region. Computational results for the three selected conformations in Figure 5 show that even though the two amino acid residues do not directly coordinate to [4Fe]_H_, the orientation of their side chains along with fluctuations of other second sphere residues influence the spin distribution within the cluster, leading to different valence isomeric forms. Specifically, the three conformers correspond to formally distinct valence isomers, in which the antiferromagnetically coupled Fe^2+^–Fe^2+^ and Fe^2.5+^–Fe^2.5+^ pairs (readily identifiable by the negative and positive spin populations, respectively, see Table S4) are located at different sides of the cubane (Figure 5C). The repositioning of Arg227 is correlated with the greatest perturbation, a reorientation of the antiferromagnetically coupled planes (model *A vs* models *B*/*C*), while comparison of models *B* and *C* shows an interchange of the two planes upon the Lys228 shift. Although it is currently not computationally feasible to establish explicit connections between these models and the axial versus rhombic EPR signals observed for [4Fe]_H_, this data strongly suggests that the reorientation of specific vicinal residues leads to valence isomer rearrangements.

### The Nuanced Effects of Second Sphere Residues Decoded by Arg227 Variants

**Figure 6:**
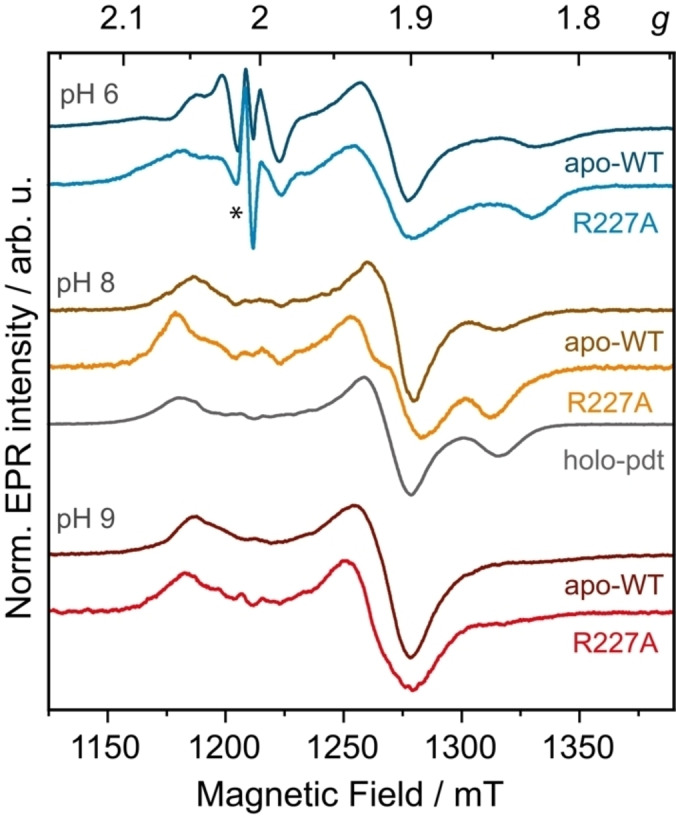
ESE‐detected EPR spectra (34 GHz, T=8/10 K) shown as pseudo‐modulations (4 mT modulation amplitude) for apo‐CrHydA1 (apo‐WT) in comparison to the R227 A variant at various pH values. The holo‐pdt variant is shown for pH 8. All variants were reduced with 5 mM NaDT. An organic radical signal is marked with *.

To explore effects of second sphere residues on the electronic structure of [4Fe]_H_, we focused on modifying Arg227. We created three variants: two neutral (R227A for alanine and R227T for threonine) and one negatively charged (R227D for aspartic acid). Considering the pH‐dependency of wild‐type EPR spectra (Figure 3B) we analyzed these variants at three distinct pH levels: 6, 8, and 9. The results showed subtle yet noticeable alterations in the EPR line shape (Figures 6 and S15).

Our attempts to simulate EPR spectra of the variants using either 4Fe−A or 4Fe−R or both species were unsuccessful. To accurately replicate the observed spectra in our simulations, it was necessary to alter both distinct species compared to the wild type. These species exhibit *g*‐values that slightly differ from those of 4Fe−A and 4Fe−R but fall within the range observed in variable‐temperature EPR spectra (Table S3).

These observations suggest that altering the second sphere Arg227 residue results in detectable changes in the electronic structure of [4Fe]_H_. They also imply that the outward and inward conformations of Arg227, as observed in our MD, do not solely account for the axial and rhombic signals observed for [4Fe]_H_. Instead, they indicate a more intricate interplay at work for valence isomer rearrangements, including the other second sphere residues as detected in the MD simulations (Figure S15).

### Mechanistic Significance of the Observed States

Despite extensive research into the [FeFe]‐hydrogenase *Cr*HydA1, the characterization of its [4Fe4S] cluster, [4Fe]_H_, has remained elusive, with previous EPR studies failing to establish universally applicable *g*‐values or comprehensive spectral simulations. This seemingly small gap in the literature presents a barrier to our understanding of the cluster's role within the enzyme and H‐cluster formation. Addressing this, our study employed an integrated approach using spectroscopy, site‐directed mutagenesis, MD simulations and QM/MM calculations. This methodology not only advances our understanding of [4Fe]_H_ but also sets a new precedent of in‐depth characterization of complex FeS clusters in biological systems.

Our comprehensive investigation of [4Fe]_H_ has led us to propose a novel ‘redox switch’ hypothesis. This concept suggests that [4Fe]_H_ exists in at least two distinct forms (4Fe−R and 4Fe−A) that dynamically interchange to regulate electron flow to the H‐cluster. This mechanism may serve as a unique adaptation in *Cr*HydA1, which lacks the accessory FeS clusters typically found in other [FeFe]‐hydrogenases. In the following discussion, we will examine the evidence supporting this hypothesis and explore its implications for our understanding of hydrogenase function and electron transfer in biological systems.

First, we identified at least two distinct EPR active species in apo‐*Cr*HydA1, designated 4Fe−R and 4Fe−A. Both species showed similar relaxation properties and average *g* values below *g*_e_≈2.0. Combined with the structural information and multi‐frequency EPR analysis, which shows the absence of magnetic interactions with other (paramagnetic) species, these data strongly indicate their origin from the same [4Fe4S]^1+^ cluster. The reliability of this conclusion is further reinforced by the consistent formation of 4Fe−A and 4Fe‐R, observed under various conditions, including changes in salt and dithionite concentration, and buffer composition. This consistency rules out the possibility of impurities as their origin.

Second, potential‐dependent measurements separated the species, revealing distinct midpoint potentials. Furthermore, pH‐dependent EPR measurements indicated that the formation of 4Fe−R is particularly sensitive to the protonation environment. These observations are critical, as they provide insights into how changes in the vicinity of [4Fe]_H_ might influence the electron transfer characteristics of the enzyme.

The variability in *g‐*values reported in the literature for [4Fe]_H_ in apo‐*Cr*HydA1, specifically the differences in *g*_3_‐values being approximately 1.85 or 1.90 (Table 1), can now be better understood through our findings. They reflect the distinct electronic structures associated with 4Fe−R and 4Fe−A, respectively. Our observations align with previous studies and explain the discrepancies noted in spectral data, emphasizing the importance of environmental factors in defining the properties of FeS clusters.

The detection of multiple EPR signatures from a single [4Fe4S]^1+^ cluster is not unique to apo‐*Cr*HydA1 but has been observed in various proteins across different species.[^66^, ^67^, ^68^, ^69^, ^70^] This suggests a common phenomenon of microheterogeneity within [4Fe4S]^1+^ clusters, which may arise from factors such as differential ligation, distinct conformations, or environmental variations influencing the cluster‘s valence isomer arrangements.[^68^, ^69^, ^70^]

Last, to elucidate the origins of several EPR signatures observed in our study, we employed MD simulations and QM/MM calculations. These computational approaches revealed conformational flexibility in the second sphere residues. Among other residues, Arg227 and Lys228 that are located proximal to [4Fe]_H_, were observed to span a wide range of conformations between ‘inward’ and ‘outward’ orientations. The transitions between outward and inward conformations of these residues, accompanied by the changes in whole second sphere and solvent environment, led us to three representative conformational states – models *A*, *B*, and *C*. We showed that such second‐sphere rearrangements correlate with unique isomeric forms of the cluster.

In order to test whether the conformational flip observed in MD simulations explain the origin of different EPR signals detected, we engineered Arg227 mutants. In all variants, we detected at least one axial and one rhombic species with slightly different *g*‐values than those observed for 4Fe−R and 4Fe−A. Our results suggest that 4Fe−R and 4Fe−A observed in [4Fe]_H_ cannot be attributed solely to conformational flip of Arg227. Rather, our findings point to a more sophisticated mechanism, where structural heterogeneity arising from a network of secondary sphere residue interactions, coupled with the rearrangement of valence isomers, leads to significant alterations in the observed EPR spectra.

This structural diversity and valence isomer rearrangements within a single cluster may also explain the complex temperature‐dependent behavior of the apo‐*Cr*HydA1 EPR line shape and the presence of at least two [4Fe]_H_ species. It is important to note, however, that this temperature‐dependent phenomenon and the presence of two distinct species do not necessarily correlate as evidenced by the experiments with plant‐type ferredoxin. This protein's only [2Fe2S] cluster exists as one species but shows similar temperature‐dependent behavior. This suggests that the temperature‐dependent behavior detected here for [4Fe]_H_ and [2Fe2S]^+^ cluster results from the presence of different valence isomers, which among other factors can arise due to structural micro and/or macro heterogeneity.

In *Cr*HydA1, assays with the Arg227 variants showed decreased activity when tested with PetF, serving as the natural electron donor. This suggested that Arg227, positioned between H‐cluster and the [2Fe2S] cluster of PetF, plays a pivotal role for functional electron transfer to H‐cluster.[^17^, ^52^] Our findings support these observations and provide a possible explanation of Arg227’s role in electron transfer and *Cr*HydA1‐PetF complex formation. The conformational flexibility of Arg227, which toggles between inward and outward orientations, appears to modulate charge of the protein surface, mediating complex formation, and alters the accessibility of [4Fe]_H_, facilitating efficient electron transfer.^[71]^ A thorough review of the literature on proteins that exhibit either an arginine[^72^, ^73^, ^74^, ^75^] or a lysine^[76]^ between two redox centers suggested to be involved in electron transfer reactions reveals a recurring theme: moderate changes in the EPR signature of the metal center due to site‐directed mutagenesis of these residues. In addition to our study, these findings underscore the critical role of positively charged second sphere residues in influencing the redox chemistry and electronic state of several metallocofactors.

FeS cluster chains play a crucial role in electron transfer for many essential enzymes. These include nitrogenase,^[77]^ carbon monoxide dehydrogenase,^[78]^ respiratory^[79]^ and photosynthetic reaction centers,^[80]^ and the majority of [FeFe]‐hydrogenases.[^77^, ^81^] These FeS cluster relays mediate and control the long‐range electron transfer to and from the enzymes’ active sites.

The efficiency and direction of catalytic reactions in these enzymes are significantly influenced by the rate of electron transfer between FeS clusters. This rate, in turn, is tuned by the redox potentials of the clusters.[^82^, ^83^] For instance, in [FeFe]‐hydrogenases from *Clostridium acetobutylicum* and *Megasphaera elsdenii* (*Me*HydA), truncation of the FeS relays (F‐clusters) reversed the catalytic bias towards H_2_ production.[^84^, ^85^] *Cr*HydA1, in contrast to these other [FeFe]‐hydrogenases, contains only the H‐cluster and lacks F‐clusters. Studies involving site‐directed mutagenesis of the coordinating cysteines of [4Fe]_H_ have shown that this cluster serves as the electron entry point. Furthermore, it modulates the H‐cluster's redox potential and controls the enzyme's catalytic bias.[^86^, ^87^]

Given that modulating redox potential influences electron transfer rates,^[88]^ we hypothesize that the observed axial and rhombic species, with distinct redox potentials, function as a ‘redox switch’ to control electron injection to the H‐cluster. This hypothesis is supported by our observation of these two species in a *Cr*HydA1 variant artificially maturated with [Fe_2_(propanedithiolate)(CO)_4_(CN)_2_]^2−^ and reduced, in which the only EPR active species is [4Fe]_H_ (holo‐pdt variant, Figure 6). Importantly, this finding suggests that the presence of these distinct [4Fe]_H_ species is intrinsic to *Cr*HydA1 and not influenced by conformational changes associated with the maturation,[^8^, ^37^, ^89^, ^90^] molecules in the empty cavity,^[8]^ or the diiron subsite.

Further evidence for this mechanism comes from site‐directed mutagenesis studies of the [4Fe]_H_ coordinating cysteines.^[87]^ These experiments revealed that different variants shift the redox potential of [4Fe]_H_ to either higher or lower values. Notably, variants that shift the redox potential to lower values appear to diminish the 4Fe−R species components in their EPR spectra, consistent with our potential dependent experiments. Additionally, the [4Fe]_H_ cluster does not exhibit such dual nature in other [FeFe]‐hydrogenases that contain F‐cluster/s.

Collectively, these data support the conclusion that [4Fe]_H_ actively participates in the redox transitions of the H‐cluster, directly linking it to the enzyme‘s functional mechanism. This ′redox switch′ could serve as a sophisticated means of regulating electron flow in *Cr*HydA1, potentially compensating for the lack of accessory FeS clusters found in other [FeFe]‐hydrogenases.

In this regard, the heterogeneity observed in [4Fe]_H_ is more than a mere spectral curiosity. Our future studies will focus on further elucidating the dynamic nature of this FeS cluster under various physiological conditions and exploring how this knowledge can be leveraged for enzyme engineering and optimization.

## Conclusion

In conclusion, our study has provided novel insights into the nature and behavior of the [4Fe4S] cluster ([4Fe]_H_) in the [FeFe]‐hydrogenase *Cr*HydA1. Through a multifaceted approach combining advanced spectroscopy, site‐directed mutagenesis, MD simulations, and QM/MM calculations, we have uncovered the existence of at least two distinct [4Fe]_H_ species, designated as 4Fe−A and 4Fe−R. Our findings reveal that these species arise from complex interplay of structural heterogeneity and valence isomer rearrangements. This heterogeneity potentially has profound implications for the function and reactivity of *Cr*HydA1. The distinct redox potentials and pH sensitivities of 4Fe−A and 4Fe−R suggest their potential role as a ‘redox switch’, dynamically controlling electron entry to the H‐cluster, the active site of the enzyme.

Furthermore, our work provides a new perspective on the previously reported variability in *g*‐values for [4Fe]_H_ in the literature, reconciling these apparent discrepancies and emphasizing the importance of considering environmental factors in FeS cluster characterization. The observed structural flexibility, particularly of residues like Arg227, offers new insights into the mechanisms of electron transfer and enzyme‐substrate interactions in *Cr*HydA1.

These results not only advance our understanding of *Cr*HydA1 but also underscore the intricate complexity of FeS clusters and their sensitivity to even subtle changes in their microenvironment.

Understanding the interplay between structure, electronic properties, and function in these systems is vital for advancing the development of bio‐inspired catalysts related to hydrogen production and providing a foundation for future studies into the fine‐tuning of FeS clusters in other biological systems.

## Supporting Information

The authors have cited additional references within the Supporting Information.[^91^, ^92^, ^93^, ^94^, ^95^, ^96^, ^97^, ^98^, ^99^, ^100^, ^101^, ^102^, ^103^, ^104^, ^105^, ^106^, ^107^, ^108^, ^109^, ^110^, ^111^, ^112^, ^113^, ^114^, ^115^, ^116^, ^117^, ^118^, ^119^, ^120^, ^121^, ^122^, ^123^]

## Conflict of Interests

The authors declare no conflict of interest.

1

## Supporting information

As a service to our authors and readers, this journal provides supporting information supplied by the authors. Such materials are peer reviewed and may be re‐organized for online delivery, but are not copy‐edited or typeset. Technical support issues arising from supporting information (other than missing files) should be addressed to the authors.

Supporting Information

## Data Availability

The data that support the findings of this study are available from the corresponding author upon reasonable request.
