# Supplementary material for: Distinct Valence States of the [4Fe4S] Cluster Revealed in the Hydrogenase CrHydA1
Source: Angew Chem Int Ed Engl. 2025 Feb 5;64(14):e202424167. doi: 10.1002/anie.202424167 (PMC11966682; doi:10.1002/anie.202424167)
Supplement: Supplementary file 1 — Supporting Information [file ANIE-64-e202424167-s001.pdf]

Angewandte Chemie
Eine Zeitschrift der Gesellschaft Deutscher Chemiker

Supporting Information

Distinct Valence States of the [4Fe4S] Cluster Revealed in the Hydrogenase *Cr*HydA1

M. Heghmanns, S. Yadav, S. Boschmann, V. R. Selve, A. Veliju, C. Brocks, T. Happe, D. A. Pantazis, M. Kasanmascheff**

Supporting Information For

Distinct Valence States of the [4Fe4S] Cluster Revealed in the Hydrogenase *Cr*HydA1

Melanie Heghmanns[a], Shalini Yadav[b], Sergius Boschmann[a], Victor R. Selve[a], Astrit Veliju[c], Claudia Brocks[c], Thomas Happe[c], Dimitrios A. Pantazis*[b], and Müge Kasanmascheff*[a]

[a] Dr. M. Heghmanns, S. Boschmann, V. R. Selve and Prof. Dr. M. Kasanmascheff
Department of Chemistry and Chemical Biology
TU Dortmund University
Otto-Hahn-Strasse 4a, 44227 Dortmund, Germany
E-mail: muege.kasanmascheff@tu-dortmund.de

[b] Dr. S. Yadav and Dr. D. A. Pantazis
Max-Planck-Institut für Kohlenforschung
Kaiser-Wilhelm-Platz 1, 45470 Mülheim an der Ruhr, Germany
E-mail: dimitrios.pantazis@kofo.mpg.de

[c] Dr. C.Brocks, A. Veliju and Prof. Dr. T. Happe
Faculty of Biology and Biotechnology, Photobiotechnology
Ruhr-University Bochum
Universitätsstrasse 150, 44801 Bochum (Germany)

Experimental section

Sample preparation

Anaerobically isolated (procedure as previously described for *Cb*A5H[1]) *Cr*HydA1 protein (apo-form), stored in Tris-HCl buffer pH 8 with 2 mM sodium dithionite (NaDT), was used. Samples were prepared in an anaerobic tent using the respective buffer with a specific pH value. Buffer exchange was performed anaerobically using Amicon® Ultra Centrifugal Filters (0.5 mL; Merck Millipore) by washing the protein 5 times. The EPR tubes were filled inside the anaerobic tent and flash-frozen in liquid nitrogen. The final enzyme concentration was 500-800 µM. The specific buffer conditions of each sample are indicated under each figure caption and summarized in Table S1. The mixed buffer was generally used for pH values 6-7 and 9. This is a 0.1 M buffer, consisting of TAPS (*N*-Tris(hydroxymethyl)-methyl-3-amino-propane sulphonic acid), HEPES (N-2-hydroxyethylpiperazine-N-2-ethane sulfonic acid), CHES (N-cyclohexyl-2-aminoethanesulfonic acid), MES (2-Morpholinoethanesulfonic acid) and sodium acetate. Tris-HCl buffer was used for the pH value of 8. Reducing buffer conditions were obtained by the addition of NaDT (5 – 200 mM).

Redox potentiometry

The redox potentiometry of apo-*Cr*HydA1 was performed anaerobically with a final concentration of 600 µM protein and 100 µM redox mediator dyes in temperature-independent (TIP) buffer (70 mM HEPES, 30 mM potassium phosphate buffer, 150 mM NaCl, and 10 % glycerol adjusted to pH 7.5)[2] at room temperature as described previously.[3] All herein-reported potentials are corrected for the standard hydrogen electrode (SHE). The potentiometry was performed in an oxidative direction, i.e., after reduction with NaDT the potential was stepwise increased with µL additions of potassium ferricyanide (FIC). Every ~50mV a sample was transferred to an EPR tube and immediately frozen in liquid nitrogen. The samples were measured via ESE-detected field-sweep experiments at Q-band frequencies with the resonator in the critically coupled mode at 10 K.

EPR spectroscopy

Q-band measurements were carried out on a Bruker ELEXSYS E580 Q-band EPR spectrometer equipped with a 150 W TWT amplifier from Applied Systems Engineering, an Oxford Instruments CF935 cryostat, and a MercuryiTC temperature controller.

Q-band ESE-detected field-sweep experiments, using a two-pulse Hahn spin-echo sequence, were acquired with a Bruker ER 5106QT-2 resonator at different temperatures in the overcoupled mode with Gaussian $\pi/2$ = 13 ns and π = 26 ns pulses with an interpulse delay of τ = 230 ns or in the critically coupled mode with rectangular $\pi/2$ = 10 ns and π = 20 ns pulses with an interpulse delay of τ = 650 ns. The shot repetition time was varied between 0.2–2 ms depending on the sample and temperature.

X-band measurements were carried out on a Bruker ELEXSYS E580 X/Q-Band EPR spectrometer equipped with a Bruker ER 4118X-MD5 resonator, a 1 kW TWT amplifier from Applied Systems Engineering for pulse measurements, an Oxford Instruments CF935 cryostat, and MercuryiTC temperature controller.

X-band ESE-detected field-sweep experiments were performed at T = 10 K with a partially overcoupled resonator using rectangular $\pi/2$ = 10 ns and π = 20 ns pulses with an interpulse delay of τ = 180 ns and a shot repetition time of 266 µs.

X-band cw EPR measurements were performed, if not stated otherwise, at various temperatures with a microwave power level of 0.018 mW, a magnetic field modulation amplitude of 8 G, a conversion time of 60 ms, and 5-10 scans.

All ESE-detected EPR spectra were normalized to the given frequency, video gain, shots per point, and number of scans. All cw EPR spectra were normalized to the given frequency, receiver gain, conversion time, square root of the power, and number of scans. The spectra were normalized, processed, and background-corrected with a polynomial function using MATLAB R2023a. Spectral simulations were performed with *EasySpin*[4] using the implemented function 'pepper'.

DEER measurements were performed on a Bruker ELEXSYS E580 X/Q-band spectrometer (see specifications above) with an ER 5106QT-2 resonator at Q-band frequencies and T = 8/10 K. A dead time free four-pulse DEER sequence ($\pi/2 - \tau_1 - \pi - (\tau_1 + t)$ - π_p - $(\tau_2 - t) - \tau_2$ - π - τ_2) was used with an interpulse delay τ_1 = 300 ns. DEER with a monochromatic pump pulse was recorded with a τ_2 of 1.1 µs, and a shot repetition time of 714 µs, while t was swept in 8 ns steps. Gaussian pulses with π_p = 32 ns and π = 74 ns were used for the pump and detect pulses, respectively. The pump pulse was offset by 90 MHz and the trace was recorded for 22 hours at 8 K. Additionally, two DEER-traces with linearly frequency swept pump pulses were recorded.[5] The first trace was recorded with a τ_2 of 3.2 µs and a t step of 22 ns. The second trace was recorded with a τ_2 of 1.59 µs and a t step of 14 ns. Both traces were recorded with a shot repetition time of 510 µs, π_p = 140 ns and, π = 48 ns with the pump pulse being linearly swept from 80 to 320 MHz. The measurements were recorded at 10 K for over 20 hours. The positions of the pump and detect pulses are marked in Figure S3A. A 16-step phase cycling scheme was applied to remove unwanted echoes.[6] Data processing and analysis were performed with DeerAnalysis2022 and Matlab2024a.[7] The resulting spectra were phase-corrected and fitted to second-order polynomial backgrounds.

Computational methods

System setup

The crystal structure of apo-*Cr*HydA1 from the organism *Chlamydomonas reinhardtii* (PDB ID: 3LX4)[8] was used to build the molecular mechanics (MM) model. The structure, resolved at 1.97 Å, was crystallized as a dimer with an RMSD difference of approximately 0.08 Å between the two units. For the MM setup, a monomeric unit was selected. Missing loops in the protein were modeled using the Modeller suite.[9] All crystallographic water molecules were retained, and missing hydrogen atoms were added using the tleap module in AMBER.[10] The FF19SB[11] force field was used for the protein amino acids. The [4Fe-4S]$(Cys)_4$ cluster, with an electronic state of [$3Fe^{2+}$:$1Fe^{3+}$], was optimized at the B3LYP/6-311G* level using CPCM solvation scheme with ORCA program. Force field parameters were incorporated into the frcmod file by the MCPB module.[12] To get the partial atomic charges of the atoms, the RESP[13,14] charge fitting scheme was employed with the Multiwfn[15] program, using the QM optimized geometry. For the non-standard organic residue ACT (acetate), the GAFF2[16] force field was used, with partial charges generated by QM optimization and fitted with RESP using Multiwfn. The resulting system was solvated with the TIP3P[17] water model up to 10 Å from the protein surface, and counter Na^+/Cl^- ions were added to maintain electrical neutrality.

MD simulation

After parameterization, the system was minimized to eliminate unfavorable contacts and to achieve geometric relaxation. This involved 5000 steps of steepest descent, followed by 5000 steps using the conjugate gradient method. The system then underwent gentle annealing for 50 ps in the NVT ensemble with protein constraints to adjust the temperature. Next, density equilibration was performed for 1 ns in the NPT ensemble, maintaining a constant temperature of 300 K and a pressure of 1.0 atm. Temperature and pressure were controlled using the Langevin[18] thermostat with a 2 ps collision frequency and the Berendsen[19] barostat with a 1 ps pressure relaxation time. This phase included MD simulation with weak restraints under periodic boundary conditions, continuing until uniform density was achieved. Once uniform density was reached, all restraints were removed. The system then underwent an additional 3 ns of equilibration followed by a 50 ns production run. The SHAKE[20] algorithm was used to constrain hydrogen atoms, and long-range electrostatic interactions were treated with the particle mesh Ewald (PME)[21] method. The simulation was performed using the GPU version of AMBER 22.[22] Analysis of the trajectory was done with the CPPTRAJ module, and trajectory visualization was carried out using VMD, with figures prepared in PYMOL.

QM/MM setup

To check the effect of different conformations observed during MD simulations on the electronic structure of the [4Fe-4S] cluster we proceeded with hybrid quantum mechanics/molecular mechanics (QM/MM) calculations on selected snapshots. The QM region includes first of all the [4Fe-4S] cluster and the cysteine residues covalently ligated with the Fe atom of the metal cluster. Additionally, from the second coordination sphere, the residues that appear most relevant for proximal conformational changes were included in the QM region (Arg227, Lys228). Water molecules that are in H-bonded interaction with [4Fe-4S] or the rg/Lys residues were also considered in the QM region (4 water molecules). For the definition of the QM region the residues are cut at the Cβ position for Cysteines and the Cδ position for Arg227 and Lys228. Hydrogens were used as link atoms when cutting through covalent bonds. The charge-shift scheme was employed at the QM/MM boundary to avoid overpolarization. The entire protein and water molecules within the 8 Å of [4Fe-4S] were included in the *active* region during QM/MM optimization, i.e. the region that is allowed to move and be optimized either with the QM or with the MM method during the QM/MM optimization. The MM atoms in the active region interact with the QM atoms through electrostatic and van der Waals interactions and the

corresponding effects were considered in the subsequent QM/MM calculations. All QM/MM calculations were performed using ORCA 5.0.[23] For the MM part we have used the ORCAFF force field and the electronic embedding scheme was used to account for the polarizing effect of the enzyme environment in the QM region. For the QM/MM geometry optimization, the QM region was computed using the hybrid B3LYP functional, accounting for scalar relativistic effects with the ZORA Hamiltonian. The relativistically adapted all-electron ZORA-TZVP basis sets were used in conjunction with the auxiliary SARC/J basis sets. All geometries have been optimized assuming an overall $[3Fe^{2+}:1Fe^{3+}(Cys)_4]^{3-}$ low spin doublet spin state, without imposing any specific spin alternation or valence localization from the outset but allowing the calculation to converge to the most favorable solution for each starting point.

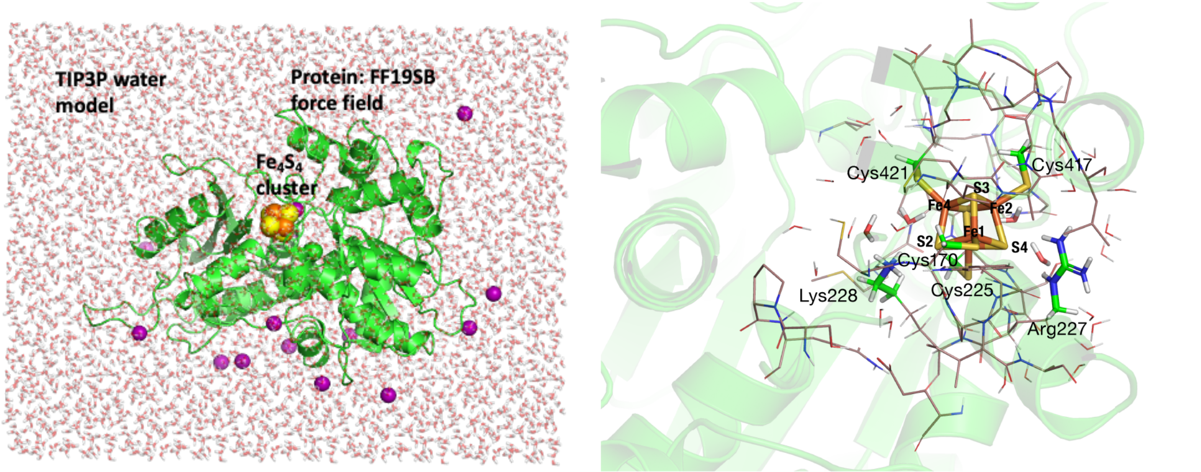

Figure S1: Left: Representation of the MM model used for the molecular dynamics simulation (purple spheres represent Na^+ ions). Right: Representation of the QM/MM model: thick sticks represent the QM region, thin sticks correspond to the complete active region that was optimized during the QM/MM optimizations..

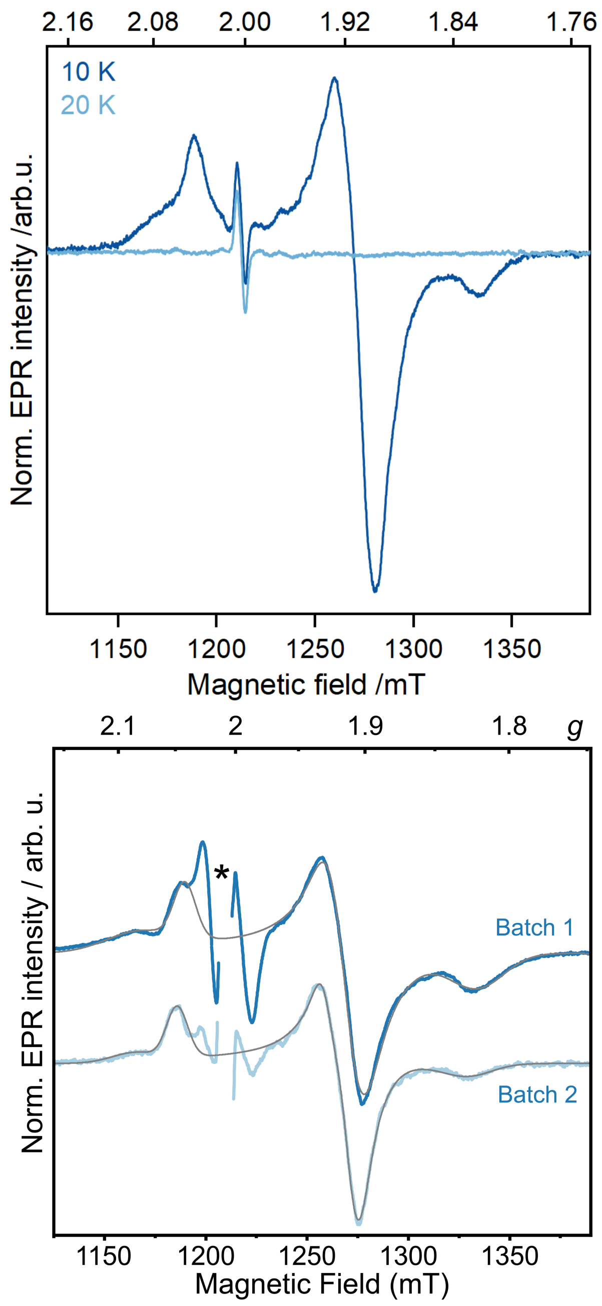

Figure S2: (Above) Comparison of pseudo-modulated (5 mT) ESE-detected EPR spectra (34 GHz) of apo-*Cr*HydA1 recorded at 10 K (dark blue trace) and 20 K (light blue trace). At 20 K, only the signal of an organic radical at $g \approx 2$, presumably arising from NaDT, is present. (Below) Comparison of pseudo-modulated (4 mT) ESE-detected EPR spectra (34 GHz) of two different apo-*Cr*HydA1 preparations and their corresponding simulations. The organic radical, marked by a *, was cut off for better readability. The samples were prepared at pH 6 by different people. The details of the simulations can be found in Table S1.

Table S1: Overview of the measured samples and parameters of associated spectral simulations performed with two different species 4Fe-A and 4Fe-R at 8/10 K with the ratio of 4Fe-A/4Fe-R. Biological replicates are marked with an * at the protein column.

Protein /mM	NaDT /mM	Buffer	pH	4Fe-A						4Fe-R						Ratio
				g-values			*g*-Strain			*g*-values			*g*-Strain			
0.8	5	0.1 M Tris-HCl	8	2.049	1.908	1.901	0.040	0.020	0.034	2.051	1.907	1.843	0.034	0.043	0.031	0.89
0.6	200	0.1 M Tris-HCl	8	2.038	1.905	1.884	0.032	0.023	0.059	2.069	1.919	1.821	0.089	0.025	0.053	2.27
0.6	10	TIP buffer	7.5	2.042	1.904	1.899	0.026	0.012	0.049	2.073	1.919	1.824	0.045	0.020	0.037	1.42
0.6	-509 mV	TIP buffer	7.5	2.042	1.905	1.904	0.023	0.018	0.043	2.064	1.920	1.830	0.033	0.018	0.042	1.17
0.6	-392 mV	TIP buffer	7.5	2.042	1.905	1.904	0.023	0.018	0.043	2.064	1.920	1.830	0.033	0.018	0.042	5.66
0.6	5	0.1 M mixed buffer	6	2.041	1.907	1.900	0.032	0.025	0.064	2.074	1.911	1.820	0.088	0.024	0.038	0.46
0.5	5	0.1 M mixed buffer	7	2.041	1.907	1.900	0.032	0.025	0.064	2.074	1.911	1.820	0.088	0.024	0.038	0.46
0.5	5	0.1 M mixed buffer + 10% glycerol	7	2.041	1.907	1.900	0.022	0.042	0.056	2.074	1.911	1.820	0.115	0.030	0.043	0.46
0.5	5	0.1 M mixed buffer	9	2.041	1.907	1.900	0.032	0.025	0.064	2.074	1.911	1.820	0.088	0.024	0.038	3.10
0.6*	5	0.1 M mixed buffer	6	2.045	1.910	1.910	0.021	0.016	0.044	2.085	1.927	1.827	0.050	0013	0.039	1.46

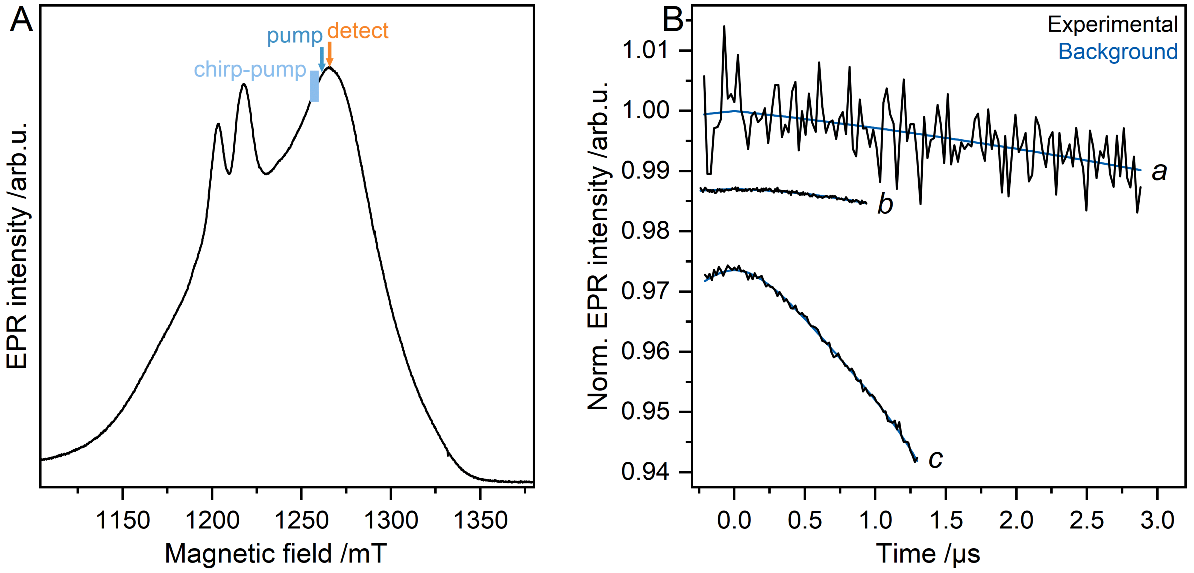

Figure S3: DEER measurements (34 GHz, T = 10/8 K) of apo-*Cr*HydA1 in mixed buffer at pH 7 and 0/10 % glycerol (chirp/monochromatic). (**A**) ESE-detected EPR spectrum with the chirp-pump (light blue area), monochromatic-pump (dark blue), and detect (orange) positions indicated. (**B**) DEER traces (black) with their respective background fits (blue) showing no modulations. The respective measurements are (***a***) chirped pump pulse (linear sweep from 80 to 320 MHz offset) τ_2 = 3.2 μs, (***b***) monochromatic pump pulse, τ_2 = 1.1 μs, (***c***) chirped pump pulse (linear sweep from 80 to 320 MHz offset) τ_2 = 1.59 μs. All traces show no modulation.

Supporting Discussion on DEER Measurements:

The short T_2 relaxation and broad spectrum limit the ability to perform DEER measurements on apo-*Cr*HydA1 resulting in low signal-to-noise and effective inversion efficiencies. We used a chirped pump pulse to mitigate the issue of low inversion efficiency. DEER spectra with and without chirped pump pulse are shown in (Figure S3B). The increase in inversion efficiency by applying chirped pump pulses is apparent when comparing traces *b* and *c* where the steepness of the background increases significantly with a chirped pump pulse.[24] None of the recorded DEER traces show any modulation indicating the absence of two integrating paramagnetic centers within a distance of 1.5 nm up to 8 nm.

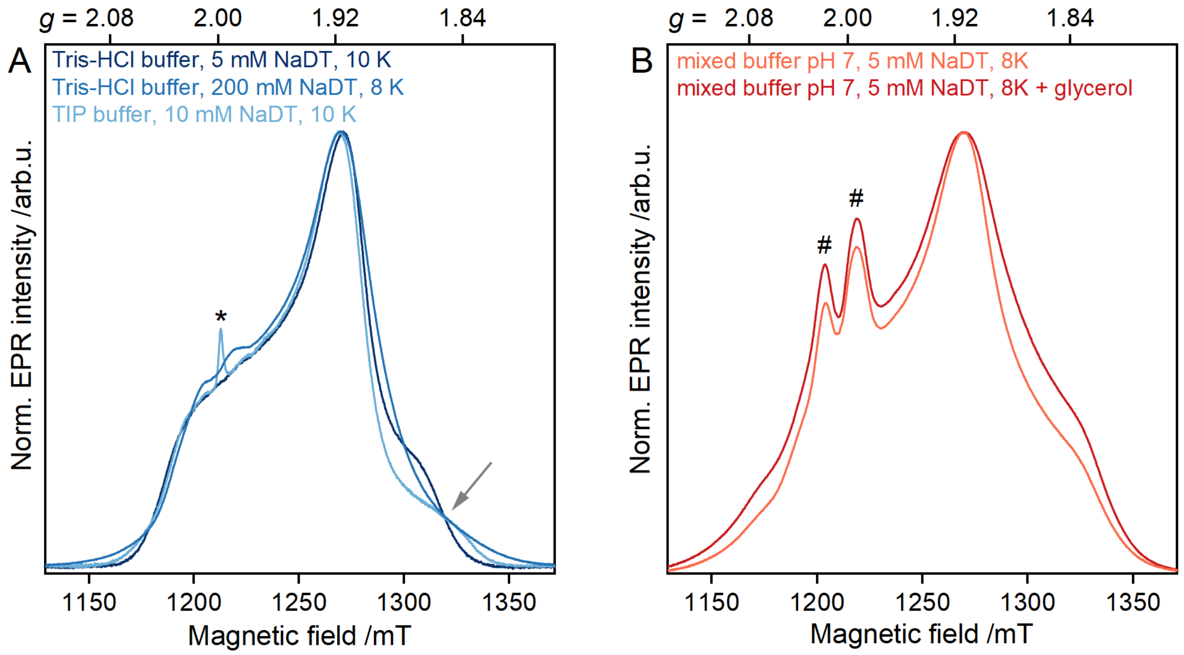

Figure S4: Comparison of ESE-detected EPR spectra (34 GHz, T = 8/10 K) of reduced apo-*Cr*HydA1 in different buffers and with varying NaDT concentrations as indicated (see Table S1 for further details). All spectra were normalized to the maximum EPR signal intensity. (**A**) A feature arising from an organic radical is marked with an asterisk. (**B**) Unknown features thought to arise from an exchange coupled organic radical are marked with hash signs (see Figure S6 for discussion).

Exchange of the buffer to a temperature-independent (TIP) buffer, used to prevent changes of pH upon freezing,[25] led to a slightly changed line shape of the rhombic species 4Fe-R at g_3. The addition of glycerol, serving as a cryoprotectant, induced an overall broadening of the line shape, which is attributed to an increase in g-strain. To investigate the influence of excessive amounts of reducing agents, the enzyme was treated with 200 mM NaDT. Again, only a small change at the g_3-position of 4Fe-R is observed. All preparations share a common cross point at $g \approx 1.85$, as indicated by the grey arrow.

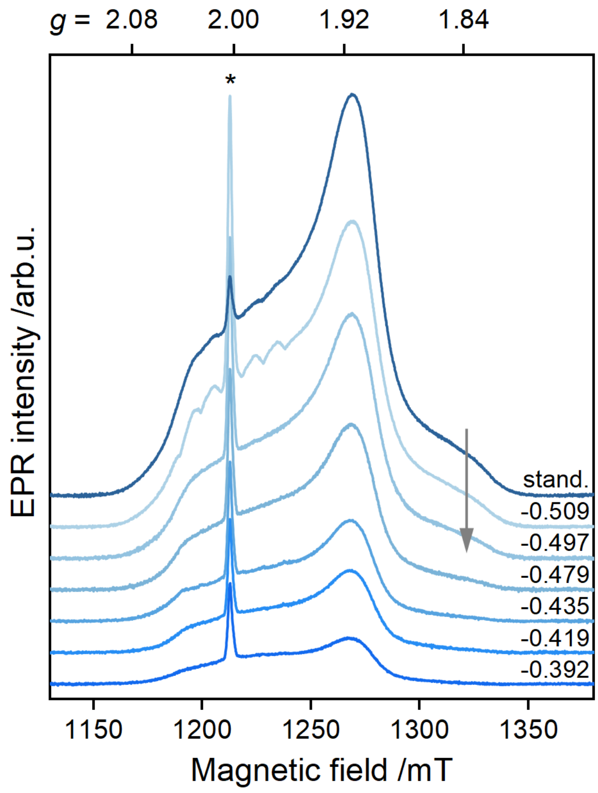

Figure S5: ESE-detected EPR spectra (34 GHz, $T = 10$ K) of the redox potentiometry samples of apo-*Cr*HydA1 taken at various potentials (in Volt) and displayed with a continuous spacing. A feature arising from an organic radical is marked with an asterisk. The spectrum denoted as standard (stand.) was reduced with 10 mM NaDT and is assumed to contain 100% reduced clusters. With increasing potential, the spectral feature around $g = 1.84$ (marked with an arrow) associated with 4Fe-R decreases in intensity.

Table S2: Relative weights of the spectral simulations of 4Fe-A and 4Fe-R used for the total spectral simulation as a function of potential *E*.

E /V	4Fe-A	4Fe-R
standard	54	46
-0.509	54	46
-0.497	57	43
-0.479	68	32
-0.435	74	26
-0.419	75	25
-0.392	85	15

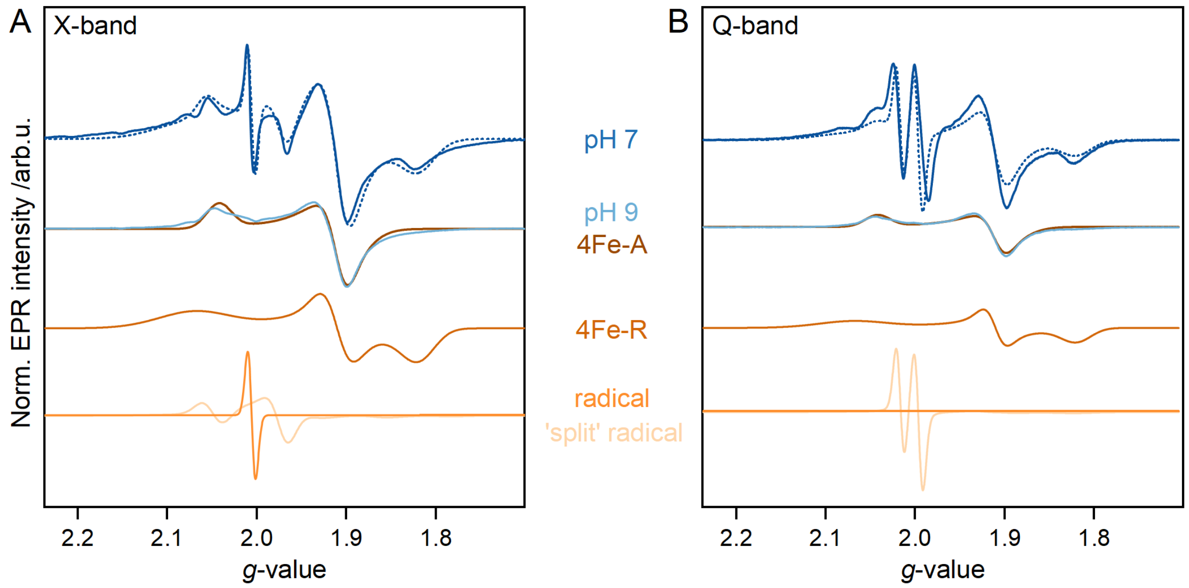

Figure S6: (**A**) Cw EPR spectra (9.5 GHz, T = 8/10 K) and (**B**) pseudo-modulated (4 mT modulation amplitude) ESE-detected EPR spectra (34 GHz, T = 10 K) of reduced apo-*Cr*HydA1 in mixed buffer at pH 7 (dark blue traces) and pH 9 (light blue traces). The total simulation (dashed blue traces) comprises the axial species 4Fe-A (brown traces), the rhombic species 4Fe-R (dark orange traces), an organic radical (orange traces), and the 'split' radical signal (beige traces). The simulation parameters are summarized in Table S1 or given in the Supporting Discussion.

Supporting Discussion on the Radical Signal

In the cw EPR spectrum (A) an organic radical species is present at $g \approx 2$. We attribute this organic radical species to a SO_2^- radical arising from NaDT in solution, having g_{iso} = 2.006 in agreement with the literature.[26] This radical species is also present in some other dithionite-reduced samples and can be isolated at elevated temperatures (compare Figure S2 or S5), which is a typical behavior for organic radicals. At Q-band frequencies (B) apo-*Cr*HydA1 samples in mixed buffer at pH 6 and pH 7 revealed two new features, marked with a hashtag in Figure 3 in the main text and Figure S6, which are absent in samples with pH ≥ 7.5. These features are centered around the *g*-value of the organic radical species. Compared to the cw X-band measurements shown in (A), those features around $g \approx 2$ appear dislocated. This frequency-dependent change in line shape is a strong indication for exchange coupling. Interestingly, temperature-dependent spectra of the pH 7 sample show that these hashtag features disappear together with 4Fe-R, indicating a similar relaxation behavior. Thus, an exchange coupling interaction between the radical and 4Fe-R is assumed. The simulation of an organic radical species coupled to 4Fe-R, termed 'split radical', can indeed reproduce the line shape at X-band and Q-band frequencies. The magnetic interaction with a relative weight of 12% was simulated with an isotropic exchange coupling of $J_{iso} = 9.4 \cdot 10^{-3}$ cm^{-1} and dipolar contributions of J_{dip} = 6.1, -2.6, -3.5 $\cdot 10^{-3}$ cm^{-1}. It has to be noted that the 'split radical' species does not influence the overall line shape of reduced apo-*Cr*HydA1 but mainly induces a splitting of the organic radical. The presence of the 'split radical' is limited to low pH samples (compare Figure S2 and S5). Even when both, 4Fe-R and the organic radical, are present at higher pH, the splitting is undetectable. Moreover, 4Fe-R is present in sample preparations lacking the organic radical. Thus, the formation of 4Fe-R is independent of the presence of the radical species. Nevertheless, the exchange interaction indicates that the dithionite radical can be located so

close to $[4Fe]_H$, that a magnetic interaction is possible. Recently, the binding of SO_2 at or near $[4Fe]_H$ was reported and our data verifies this assignment.[27] The biological implication of this interaction is currently unknown.

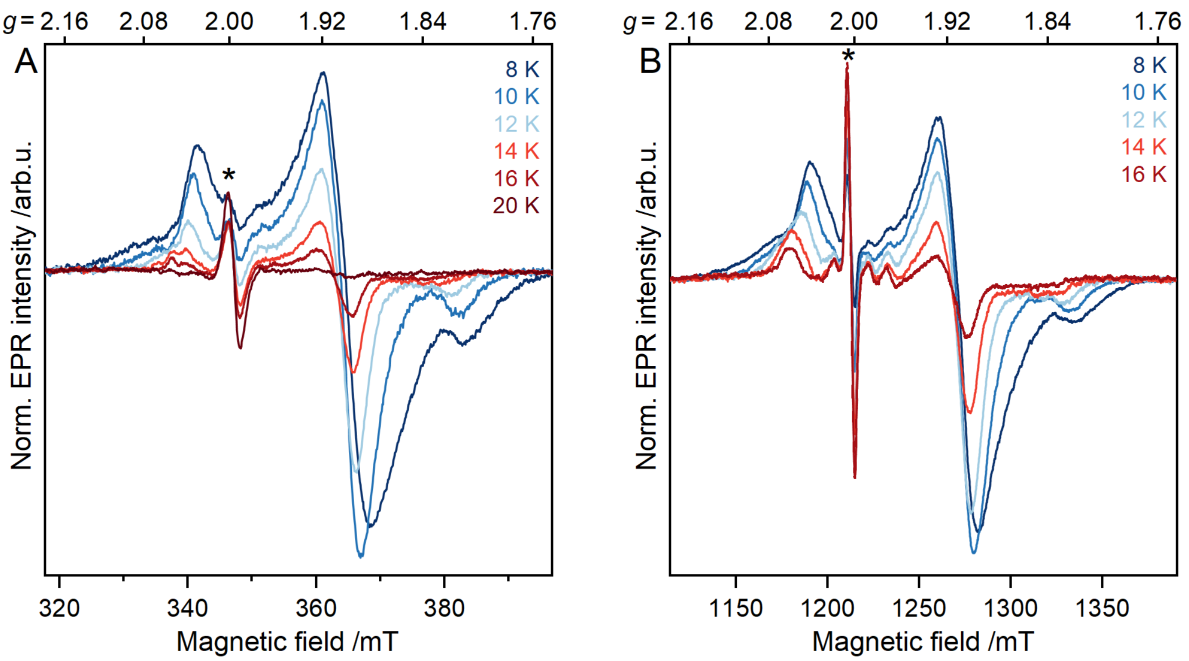

Figure S7: Temperature-dependent, pseudo-modulated ((A): 1.4 mT, (B): 5 mT) ESE-detected EPR spectra of apo-*Cr*HydA1 in TIP buffer at pH 7.5 reduced with 10 mM NaDT recorded at (**A**) 9.7 GHz and (**B**) 34 GHz. An organic radical signal is marked with asterisks. At 20 K, the organic radical is the only species detectable. Upon increasing temperature, the EPR line shape changes. This presumably arises from multiple overlapping species with distinct relaxation behaviour (see also Figure S11).

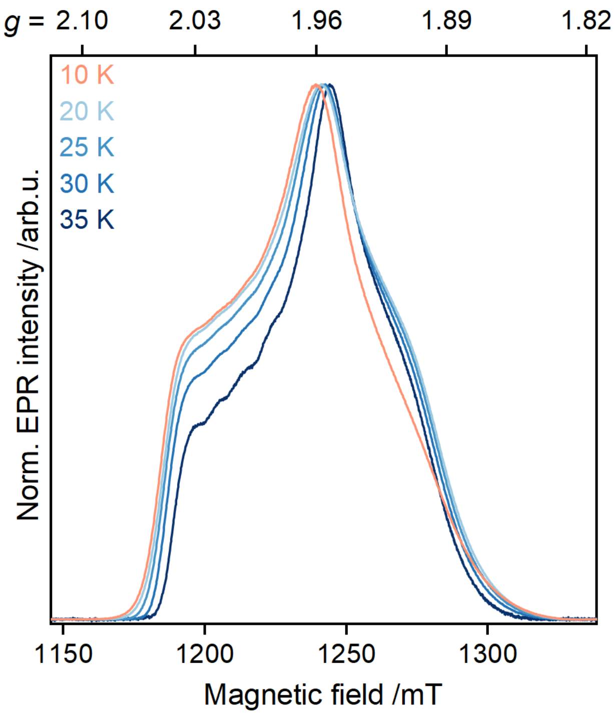

Figure S8: Temperature-dependent ESE-detected EPR spectra (34 GHz) of a reduced [2Fe2S] cluster-containing ferredoxin. All canonical *g*-value positions exhibit line shape changes upon increasing temperature.

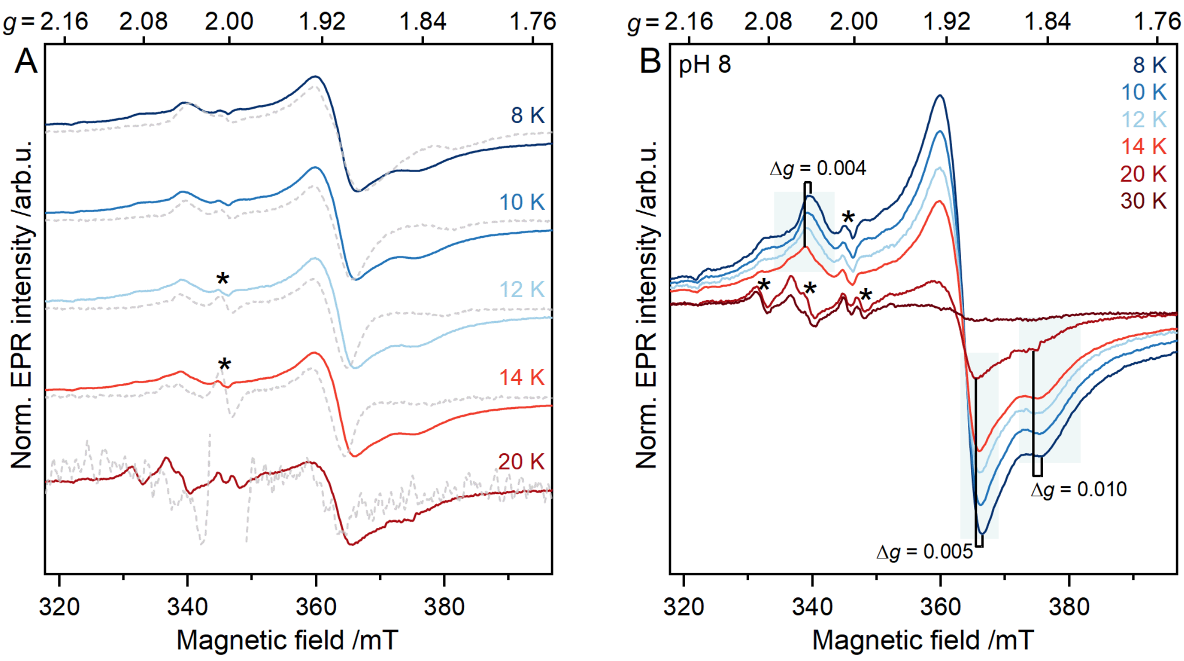

Figure S9: **(A)** Comparison of temperature-dependent, pseudo-modulated (1.4 mT) ESE-detected (dashed traces) and cw (0.8 mT modulation amplitude) EPR spectra (solid traces, 9.7 GHz) of reduced apo-*Cr*HydA1 in Tris-HCl or TIP buffer at pH 8. **(B)** Cw spectra from (A) plotted on top of each other for better comparability covering a larger temperature range with the changes in line shape indicated as Δ*g*. Compared to the pulse spectra, the change is virtually absent from the cw spectra. Background signals or impurities are marked with asterisks.

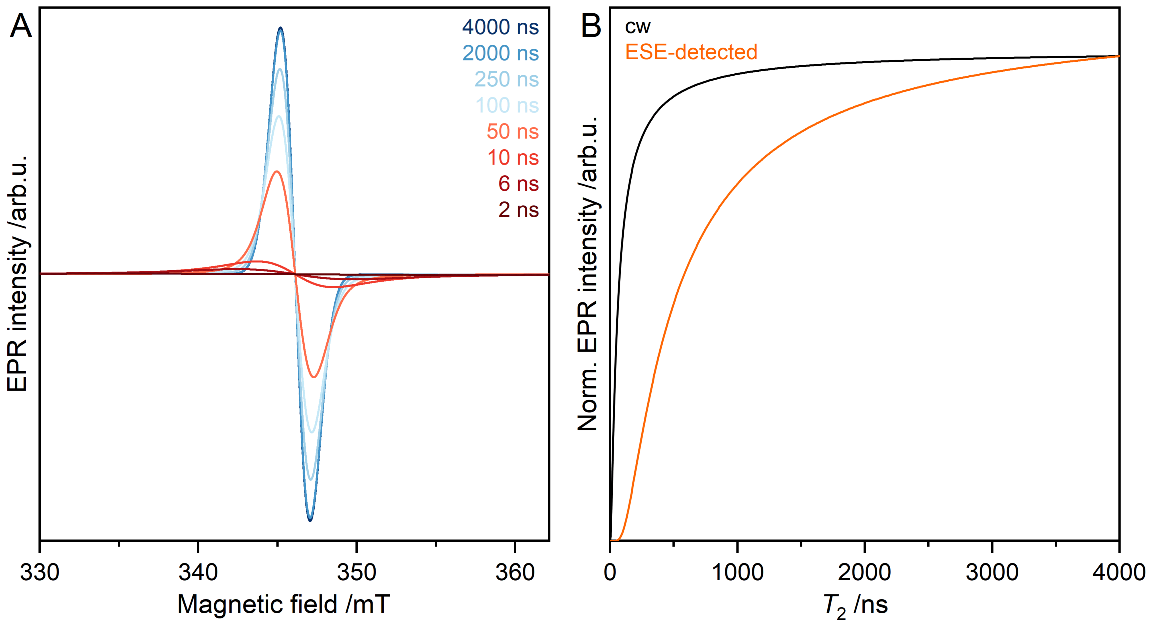

Figure S10: **(A)** Simulated cw EPR spectra (9.7 GHz, g = 2.002) for different T_2 values at a modulation amplitude of 0.8 mT. The spectra were constructed as Lorentzian shapes with a full width at half maximum (FWHM) of $2/T_2$ and further convoluted with a Gaussian shape with a FWHM of 60 MHz using the *EasySpin* function 'convspec'.[28] This value was chosen to simulate the effect of g-strain and is based on the smallest strain used in our simulations. **(B)** Comparison of the relative signal strengths (measured as a peak-to-peak intensity) of cw and ESE-detected EPR spectra as a function of T_2. The spectra were calculated as described in (A) with a modulation amplitude of 1.4 mT used for the ESE-detected EPR spectra. To include the drop-off in signal intensity due to relaxation during evolution for the ESE-detected EPR spectra, exp $[-\left(\frac{2\tau}{T_2}\right)]$ with a τ of 180 ns was used. The factor from the exponential decay was multiplied with the peak-to-peak intensity.

Supporting Discussion on cw *versus* pulsed EPR spectroscopy

Compared to non-saturating cw EPR, the relative intensities of species in ESE-detected EPR show a strong dependency on their respective spin-lattice (T_1) and transverse (T_2) relaxation times.[29,30] This dependence has been exploited in pulsed experiments specifically designed to separate species, such as relaxation-filtered hyperfine (REFINE) spectroscopy.[31] In pulsed EPR, T_1-based filtering occurs when the repetition time between pulse sequences is short in comparison to T_1. In this case, spins with longer T_1 are not able to return to their equilibrium magnetization before the next pulse sequence and are therefore suppressed.[31,32] The T_2 relaxation describes interactions within the spin system during the magnetization fanning out in the x/y-plane. In the simplest case the decay is described as by an exponential function (exp $[-\left(\frac{2\tau}{T_2}\right)]$) for an evolution time of 2 τ. For ESE-detected EPR spectra this means that the signal intensity will drop with an increasing τ.[33] In non-saturating cw EPR spectroscopy, T_1 and T_2 only influence the line width in the form of homogenous broadening. Usually, being the faster relaxation mechanism, T_2 can often be taken as the dominant factor for homogenous broadening.[28] Figure S11 depicts the influence T_2 has on the relative signal strengths for pulsed and cw EPR. As T_2 decreases, we would expect a gradual change in signal strength in pulsed EPR. In contrast, for cw EPR only a small change in signal strength, followed by a rapid decline at very short T_2 is expected. As shown, the line shape of the cw EPR spectra is virtually constant up to 14 K (Figure S10), followed by large changes with some species being no longer detected. Contrarily, the ESE-detected spectra change more gradually, in line with the exponential dependence of the ESE intensity on T_2 (Figure S11B).

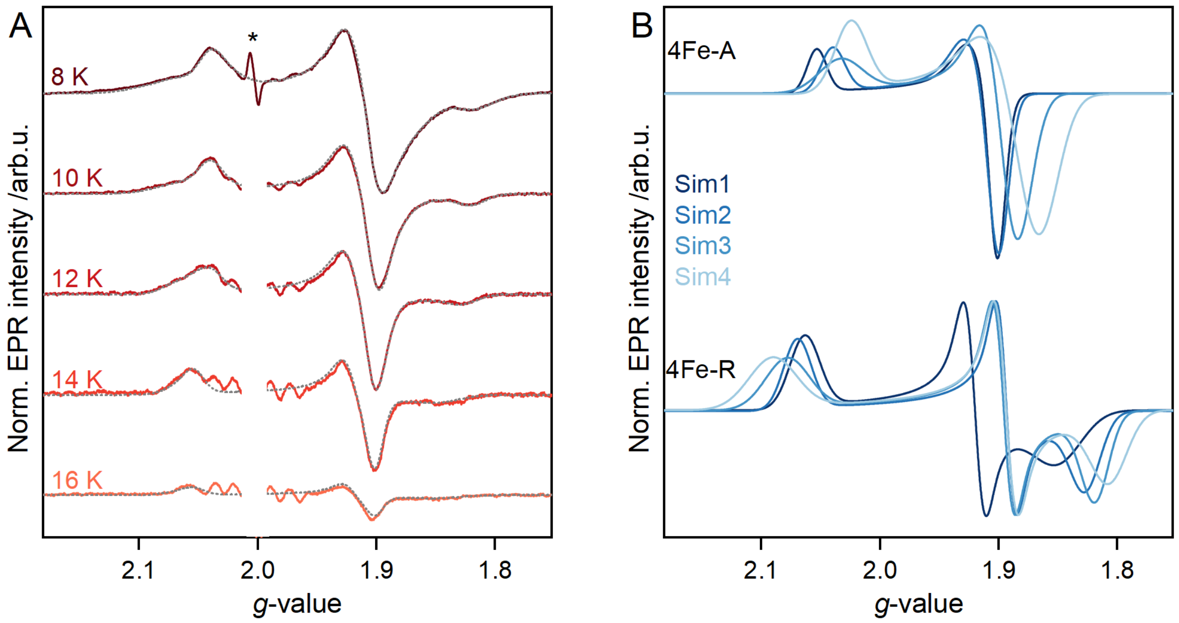

Figure S11: (**A**) Temperature-dependent, pseudo-modulated (5 mT) ESE-detected EPR spectra (34 GHz) of apo-*Cr*HydA1 in TIP buffer at pH 7.5 reduced with 10 mM NaDT recorded at distinct temperatures (colored traces) and respective total simulations (grey dotted traces). An organic radical signal is marked with an asterisk and cut out for better visibility at temperatures above 8 K. (**B**) Overview of the single systems (four for each species 4Fe-A and 4Fe-R, respectively) used to construct the total simulations at each temperature. The simulation parameters are given below.

Table S3: Simulation parameters and weight percentages for the total simulations of reduced apo-*Cr*HydA1 in TIP buffer (pH 7.5) measured at distinct temperatures at Q-band frequencies shown in Figure S12. The average simulation parameters, avg, were derived from the average of four different systems for each species 4Fe-A and 4Fe-R.

	g-value			*g*-Strain			Weight at given temperature / %				
System	g_1	g_2	g_3	gS_1	gS_2	gS_3	8 K	10 K	12 K	14 K	16 K
4Fe-A1	2.053	1.914	1.903	0.018	0.029	0.014	1	2	12	45	35
4Fe-R1	2.063	1.919	1.848	0.029	0.016	0.048	7	11	16	55	65
4Fe-A2	2.040	1.915	1.901	0.023	0.030	0.018	29	42	45		
4Fe-R2	2.070	1.893	1.827	0.027	0.016	0.030	8	4	27		
4Fe-A3	2.034	1.898	1.886	0.043	0.031	0.032	13	16			
4Fe-R3	2.079	1.896	1.819	0.043	0.017	0.027	8	25			
4Fe-A4	2.024	1.891	1.867	0.031	0.048	0.037	16				
4Fe-R4	2.091	1.894	1.807	0.047	0.018	0.035	19				
4Fe-A avg	2.038	1.904	1.889	0.029	0.034	0.025					
4Fe-R avg	2.076	1.900	1.825	0.036	0.017	0.035					
4Fe-A range	2.053	1.915	1.903	0.043	0.048	0.037					
	2.024	1.891	1.867	0.018	0.029	0.014					
4Fe-R range	2.091	1.919	1.848	0.047	0.018	0.048					
	2.063	1.893	1.807	0.027	0.016	0.027					

Supporting Discussion on Simulation Parameters from Figure S12

We attempt to explain the observed line shape changes by proposing that spectra of reduced apo-*Cr*HydA1 are the sum of multiple species whose relaxation times change differently with temperature. We are aware, that inhomogeneous broadening and anisotropic relaxation effects may play a role in the distinct distribution,[34] however, they are unlikely the reason for such a change in line shape,. i.e.,*g*-values. Here, we present a possible combination of species, similar as for $[4Fe4S]^{3+}$ clusters,[35] which enabled us to accurately reproduce the EPR spectra of apo-*Cr*HydA1 at various temperatures. We began our simulations with the 16 K spectrum with an axial and rhombic species (4Fe-A1 and 4Fe-R1, respectively), whose relative weights changed at 14 K. At 12 K, the addition of two more species, one axial and one rhombic, was necessary. Simultaneously, the simulation parameters of 4Fe-A1 and 4Fe-R1 were kept constant. We continued in this manner until 8 K, yielding a total of eight distinct species. The values of 4Fe-A are in the range of g_1 = 2.053-2.024, g_2 = 1.915-1.891, and g_3 = 1.903-1.867, and of 4Fe-R in the range of g_1 = 2.091-2.063, g_2 = 1.919-1.893, and g_3 = 1.848-1.807. Thus, different ranges were achieved for g_1 and g_3 of 4Fe-A and 4Fe-R, respectively. The ratio including all simulation species associated with 4Fe-A and 4Fe-R changed between 1.4 (8 K) and 0.5 (16 K) (Figure S13).

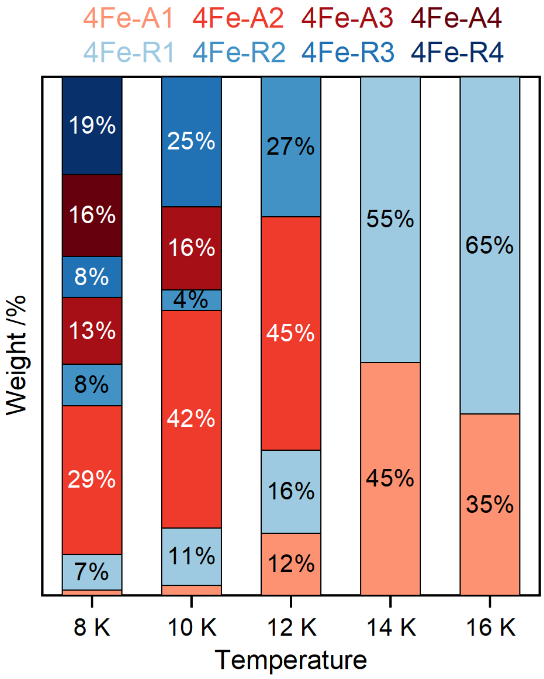

Figure S12: Weight percentages of each species used for the simulations shown in Figure S12.

As demonstrated in Figures 2-3 and Table S1, spectral simulations of apo-*Cr*HydA1 at low temperatures can also be achieved with two distinct species. However, the spectral line shape cannot be fully reproduced. As explained in the main text, the complexity of this system introduces several variables, making a definitive analysis challenging. Nevertheless, our data intends to show, that it is impossible to define one set of *g*-values for the EPR spectra of the $[4Fe4S]^{+}$ cluster in apo-*Cr*HydA1 at various temperatures. Thus, future research needs to consider the buffer conditions, measurement parameters, and methods for comparability of recorded species and *g*-values. Eventually, a systematic study including several $[4Fe4S]^{+}$ clusters from different proteins might help to elucidate their complex electronic structure and link certain features to their spectral properties, as previously performed for $[3Fe4S]^{+}$ and $[4Fe4S]^{3+}$ clusters.[35,36]

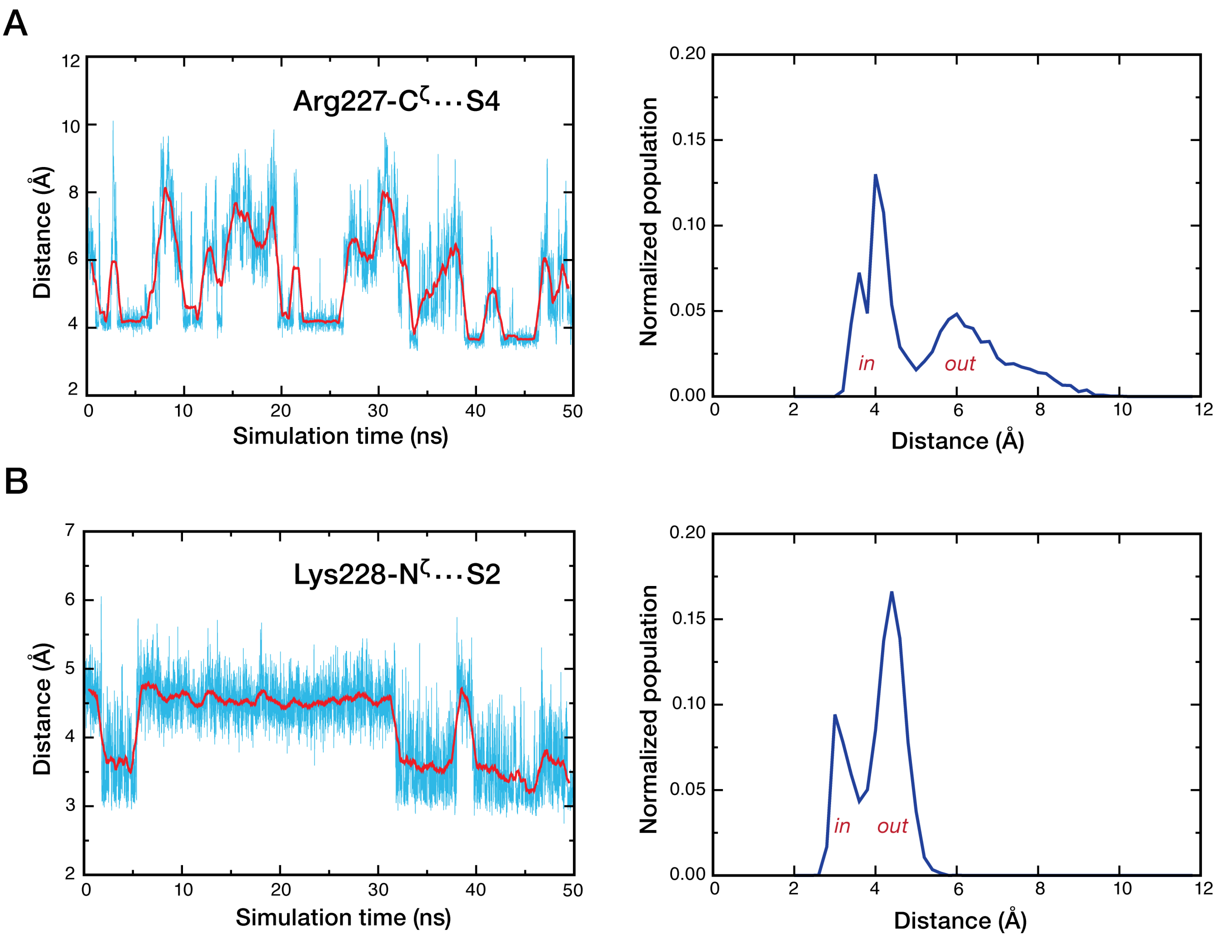

Figure S13: (**A**) Distance plot between the Arg227 C^{ζ} atom of the guanidinium and the S4 bridge of the $[4Fe]_H$ cluster in the MD simulations (left) and normalized population (right) showing the distinct distributions of inward and outward orientations of Arg227; (**B**) Distance plot between the Lys228 N^{ζ} atom and the S2 bridge of the $[4Fe]_H$ cluster in the MD simulations (left) and normalized population (right).

Table S4: Spin populations computed from QM/MM calculations for the three combinations of Arg227 and Lys228 orientations presented in Figure 4 of the main text.

	**Model A**	**Model B**	**Model C**
Fe1	3.734	3.812	-3.604
Fe2	-3.607	-3.624	3.699
Fe3	3.717	-3.589	3.752
Fe4	-3.610	3.687	-3.631
S1	0.067	0.016	0.234
S2	0.259	0.264	0.101
S3	0.082	0.224	0.068
S4	0.192	0.080	0.269

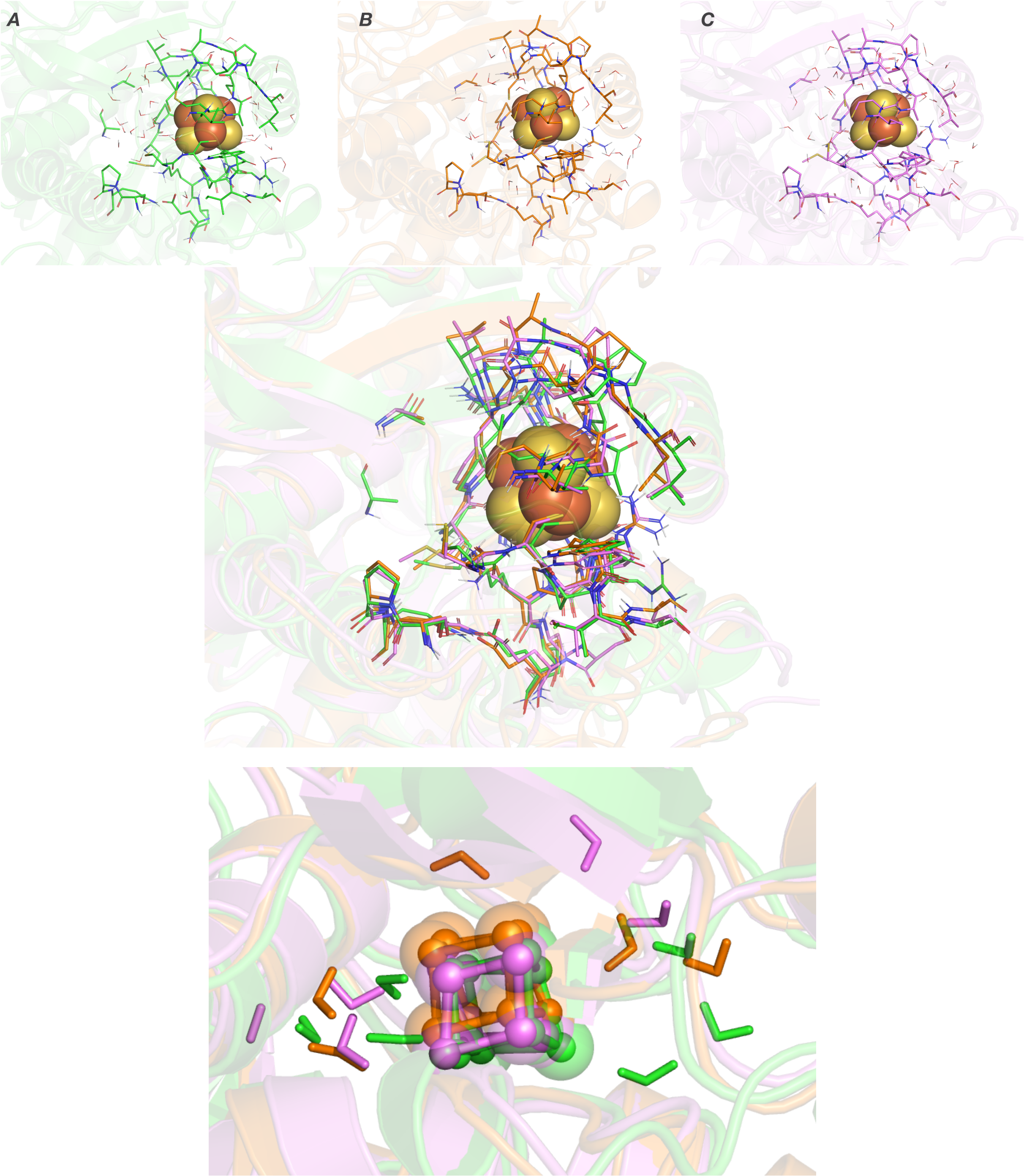

Figure S14: (**Above**) The three QM/MM models with the complete optimized region around $[4Fe]_H$ and the optimized water molecules. (**Middle**) Overlaid depiction of three models without waters demonstrating how each model has general differences in the second coordination sphere that extend far beyond Arg227 and Lys228. (**Bottom**) Overlaid depiction of three models showing the structural changes of the $[4Fe]_H$ cluster and the surrounding water-molecules.

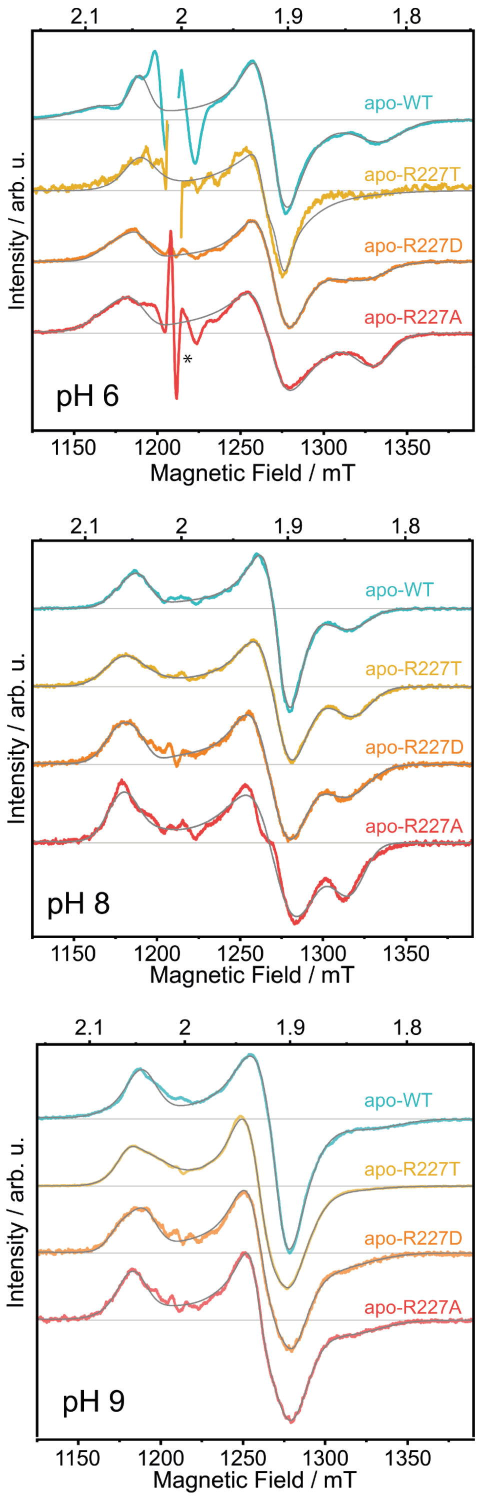

Figure S15: ESE-detected EPR spectra (34 GHz, T = 8/10 K) shown as pseudo-modulations (4 mT modulation amplitude) for apo-CrHydA1 (apo-WT) in comparison to distinct Arg227 variants at various pH values. The corresponding simulations are shown with grey lines. All variants were reduced with 5 mM NaDT. An organic radical signal is marked with *.

References

[1] M. Heghmanns, A. Rutz, Y. Kutin, V. Engelbrecht, M. Winkler, T. Happe, M. Kasanmascheff, *Chem. Sci.* **2022**, *13*, 7289–7294.

[2] N. Le Breton, J. J. Wright, A. J. Y. Y. Jones, E. Salvadori, H. R. Bridges, J. Hirst, M. M. Roessler, *J. Am. Chem. Soc.* **2017**, *139*, 16319–16326.

[3] M. Heghmanns, A. Günzel, D. Brandis, Y. Kutin, V. Engelbrecht, M. Winkler, T. Happe, M. Kasanmascheff, *Biophys. Rep.* **2021**, *1*, 100016.

[4] S. Stoll, A. Schweiger, *J. Magn. Reson.* **2006**, *178*, 42–55.

[5] A. Doll, S. Pribitzer, R. Tschaggelar, G. Jeschke, *J. Magn. Reson.* **2013**, *230*, 27–39.

[6] C. E. Tait, S. Stoll, *Phys. Chem. Chem. Phys.* **2016**, *18*, 18470–18485.

[7] G. Jeschke, V. Chechik, P. Ionita, A. Godt, H. Zimmermann, J. Banham, C. R. Timmel, D. Hilger, H. Jung, *Appl. Magn. Reson.* **2006**, *30*, 473–498.

[8] D. W. Mulder, E. S. Boyd, R. Sarma, R. K. Lange, J. A. Endrizzi, J. B. Broderick, J. W. Peters, *Nature* **2010**, *465*, 248–251.

[9] A. Šali, T. L. Blundell, *J. Mol. Biol.* **1993**, *234*, 779–815.

[10] D. A. Case, H. M. Aktulga, K. Belfon, I. Ben-Shalom, S. R. Brozell, D. S. Cerutti, T. E. Cheatham Iii, V. W. D. Cruzeiro, T. A. Darden, R. E. Duke, **2021**.

[11] C. Tian, K. Kasavajhala, K. A. A. Belfon, L. Raguette, H. Huang, A. N. Migues, J. Bickel, Y. Wang, J. Pincay, Q. Wu, C. Simmerling, *J. Chem. Theory Comput.* **2020**, *16*, 528–552.

[12] P. Li, K. M. Jr. Merz, *J. Chem. Inf. Model.* **2016**, *56*, 599–604.

[13] C. I. Bayly, P. Cieplak, W. Cornell, P. A. Kollman, *J. Phys. Chem.* **1993**, *97*, 10269–10280.

[14] W. D. Cornell, P. Cieplak, C. I. Bayly, P. A. Kollman, *J. Am. Chem. Soc.* **1993**, *115*, 9620–9631.

[15] T. Lu, F. Chen, *J. Comput. Chem.* **2012**, *33*, 580–592.

[16] D. Vassetti, M. Pagliai, P. Procacci, *J. Chem. Theory Comput.* **2019**, *15*, 1983–1995.

[17] W. L. Jorgensen, J. Chandrasekhar, J. D. Madura, R. W. Impey, M. L. Klein, *J. Chem. Phys.* **1983**, *79*, 926–935.

[18] J. A. Izaguirre, D. P. Catarello, J. M. Wozniak, R. D. Skeel, *J. Chem. Phys.* **2001**, *114*, 2090–2098.

[19] H. J. C. Berendsen, J. P. M. Postma, W. F. van Gunsteren, A. DiNola, J. R. Haak, *J. Chem. Phys.* **1984**, *81*, 3684–3690.

[20] J.-P. Ryckaert, G. Ciccotti, H. J. C. Berendsen, *J. Comput. Phys.* **1977**, *23*, 327–341.

[21] T. Darden, D. York, L. Pedersen, *J. Chem. Phys.* **1993**, *98*, 10089–10092.

[22] R. Salomon-Ferrer, A. W. Götz, D. Poole, S. Le Grand, R. C. Walker, *J. Chem. Theory Comput.* **2013**, *9*, 3878–3888.

[23] F. Neese, *WIREs Comput. Mol. Sci.* **2022**, *12*, e1606.

[24] M. Pannier, S. Veit, A. Godt, G. Jeschke, H. W. Spiess, *J. Magn. Reson.* **2011**, *213*, 316–325.

[25] N. A. Sieracki, H. J. Hwang, M. K. Lee, D. K. Garner, Y. Lu, *Chem Commun* **2008**, 823–825.

[26] R. G. Rinker, T. P. Gordon, D. M. Mason, W. H. Corcoran, *J Am Chem Soc* **1958**, *17*, 302.

[27] M. A. Martini, O. Rüdiger, N. Breuer, B. Nöring, S. DeBeer, P. Rodríguez-Maciá, J. A. Birrell, *J. Am. Chem. Soc.* **2021**, *143*, 18159–18171.

[28] M. Brustolon, E. Giamello, *Electron Paramagnetic Resonance: A Practitioner's Toolkit*, John Wiley & Sons, Inc., Hoboken, NJ, USA, **2008**.

[29] G. R. Eaton, S. S. Eaton, D. P. Barr, R. T. Weber, *Quantitative EPR*, Springer, Vienna, **2010**.

[30] J. A. Weil, J. R. Bolton, *Electron Paramagnetic Resonance: Elementary Theory and Practical Applications*, Wiley & Sons Ltd, Hoboken, N.J, **2007**.

[31] T. Maly, T. F. Prisner, *J. Magn. Reson.* **2004**, *170*, 88–96.

[32] I. Kaminker, M. Florent, B. Epel, D. Goldfarb, *J. Magn. Reson.* **2011**, *208*, 95–102.

[33] S. R. Eaton, G. R. Eaton, L. J. Berliner, *Biomedical EPR - Bart B: Methodology, Instrumentation and Dynamics*, **2005**.

[34] W. R. Hagen, *J. Biol. Inorg. Chem.* **2018**, *23*, 623–634.

[35] A. H. Priem, A. A. K. Klaassen, E. J. Reijerse, T. E. Meyer, C. Luchinat, F. Capozzi, W. R. Dunham, W. R. Hagen, *J. Biol. Inorg. Chem.* **2005**, *10*, 417–424.
[36] J. Telser, H.-I. Lee, B. M. Hoffman, *J. Biol. Inorg. Chem.* **2000**, *5*, 369–380.
